# CDKL5 regulates p62-mediated selective autophagy and confers protection against neurotropic viruses

**DOI:** 10.1172/JCI168544

**Published:** 2024-01-02

**Authors:** Josephine W. Thinwa, Zhongju Zou, Emily Parks, Salwa Sebti, Kelvin Hui, Yongjie Wei, Mohammad Goodarzi, Vibha Singh, Greg Urquhart, Jenna L. Jewell, Julie K. Pfeiffer, Beth Levine, Tiffany A. Reese, Michael U. Shiloh

**Affiliations:** 1 Department of Internal Medicine,; 2 Department of Microbiology,; 3 Department of Cell Biology, and; 4 Howard Hughes Medical Institute, University of Texas Southwestern Medical Center, Dallas, Texas, USA.; 5 Center for iPS Cell Research and Application, Kyoto University, Kyoto, Japan.; 6 Cancer Research Institute, Guangzhou Medical University, Guangzhou, China.; 7 Department of Immunology, and; 8 Department of Molecular Biology, University of Texas Southwestern Medical Center, Dallas, Texas, USA.

**Keywords:** Infectious disease, Virology, Autophagy, Cellular immune response, Innate immunity

## Abstract

Virophagy, the selective autophagosomal engulfment and lysosomal degradation of viral components, is crucial for neuronal cell survival and antiviral immunity. However, the mechanisms leading to viral antigen recognition and capture by autophagic machinery remain poorly understood. Here, we identified cyclin-dependent kinase–like 5 (CDKL5), known to function in neurodevelopment, as an essential regulator of virophagy. Loss-of-function mutations in CDKL5 are associated with a severe neurodevelopmental encephalopathy. We found that deletion of CDKL5 or expression of a clinically relevant pathogenic mutant of CDKL5 reduced virophagy of Sindbis virus (SINV), a neurotropic RNA virus, and increased intracellular accumulation of SINV capsid protein aggregates and cellular cytotoxicity. *Cdkl5*-knockout mice displayed increased viral antigen accumulation and neuronal cell death after SINV infection and enhanced lethality after infection with several neurotropic viruses. Mechanistic studies demonstrated that CDKL5 directly binds the canonical selective autophagy receptor p62 and phosphorylates p62 at T269/S272 to promote its interaction with viral capsid aggregates. We found that CDKL5-mediated phosphorylation of p62 facilitated the formation of large p62 inclusion bodies that captured viral capsids to initiate capsid targeting to autophagic machinery. Overall, these findings identify a cell-autonomous innate immune mechanism for autophagy activation to clear intracellular toxic viral protein aggregates during infection.

## Abstract 2

CDKL5, a kinase crucial for neurodevelopment, also regulates virophagy in mouse models by regulating autophagic degradation of viral capsids and limiting infection-induced neuronal cell death.

## Graphical abstract

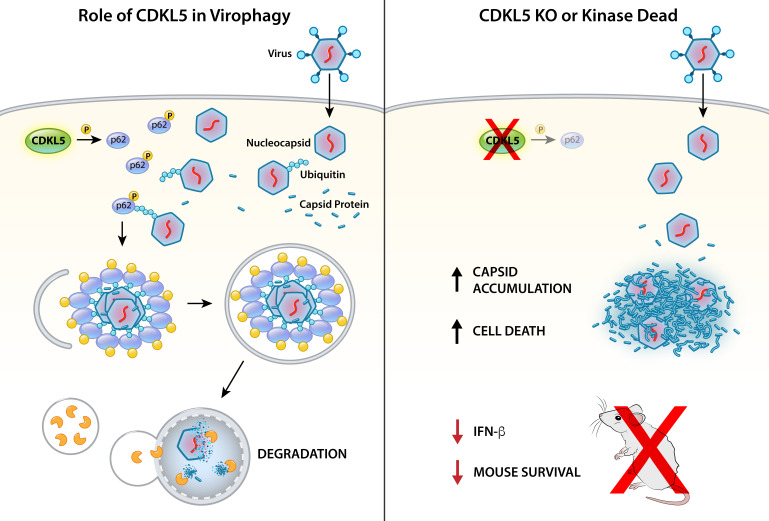

## Introduction

Upon infection, host cells become virus production factories, leading to accumulation of large quantities of viral proteins. When viral protein accumulation overwhelms cellular degradative capabilities, toxic protein aggregates form, leading to cell death (1). Host cell survival thus depends not only on preventing viral replication, but also on eliminating viral debris. Autophagy is a cell-autonomous process for cellular sequestration of unwanted cytoplasmic components such as damaged organelles, protein aggregates, and invading pathogens into double-lipid-membrane vesicles for subsequent lysosomal degradation (2, 3). Cargo uptake occurs either nonselectively or selectively based on cargo recognition by specific autophagy receptors. The selective autophagic degradation of infectious cargo is called xenophagy. Viral xenophagy, or simply virophagy, targets viral antigens and particles to the autophagosome for degradation and plays an important role in cell-autonomous innate immunity. Several selective autophagy receptors participate in xenophagy, including p62/SQSTM1**,** OPTN, NDP52, NBR1, and TAX1BP1 (4). However, to date, the induction and regulation mechanisms of receptor-cargo recognition that initiate virophagy remain poorly understood.

Depending on the type of cell or virus, autophagy can promote or disrupt viral replication. Neurons, a nonrenewable cell population, rely heavily on virophagy for protection against viral infection. For example, virophagy is critical for the antiviral response and host survival during SINV infection (5, 6). SINV is a positive-sense RNA alphavirus that causes murine viral encephalitis in a manner analogous to human disease caused by other arthropod-borne viruses. Overexpression of an essential autophagy gene, *Beclin1*, decreases SINV replication and protects in vivo neuronal cells from apoptosis, leading to enhanced mouse survival after lethal intracerebral infection (6). Conversely, genetic disruption of another essential autophagy gene, *Atg5*, causes SINV capsid protein accumulation in both cultured cell lines and murine brain tissue, with associated neuronal cell death (5). Thus, virophagy plays a cytoprotective role in neurons in part by preventing virus-induced cell death (1).

Although the involvement of essential autophagic machinery in viral pathogenesis is well established, our knowledge of host factors that regulate the selective targeting of autophagic machinery to viral antigens is quite limited. Recently, a genome-wide image-based siRNA screen in HeLa cells infected with SINV or herpes simplex virus type 1 (HSV-1) identified over 200 genes as putative regulators of virus-induced autophagy, including the serine/threonine kinase cyclin-dependent kinase–like 5 (CDKL5) (7). We chose to investigate the potential autophagic function of CDKL5, as it is widely expressed, with highest expression in neurons where it functions in neuronal morphogenesis (8, 9) and has no prior established role in autophagy. Notably, human *CDKL5* mutations result in a severe neurodevelopmental encephalopathy through a poorly understood mechanism (8–10). Thus, identifying a critical role for CDKL5 in triggering autophagy has implications for our understanding of both the molecular mechanisms of viral antigen clearance and neuronal homeostasis.

Here, we demonstrate that CDKL5 is a key initiator of virus-triggered autophagy and protects neuronal cells in vivo and mice from death during SINV, HSV-1, and chikungunya virus (CHIKV) infections. A known human pathogenic mutation of CDKL5 that disrupts its kinase activity failed to rescue autophagy activation and SINV capsid protein accumulation, indicating the necessity of CDKL5 kinase activity for autophagic degradation of capsid proteins. We further demonstrate that CDKL5 directly phosphorylates p62 at T269/S272, which is essential for p62 to capture viral capsid aggregates.

## Results

### Basal and virus-induced autophagy requires CDKL5 in a kinase-dependent manner.

**Figure 1:**
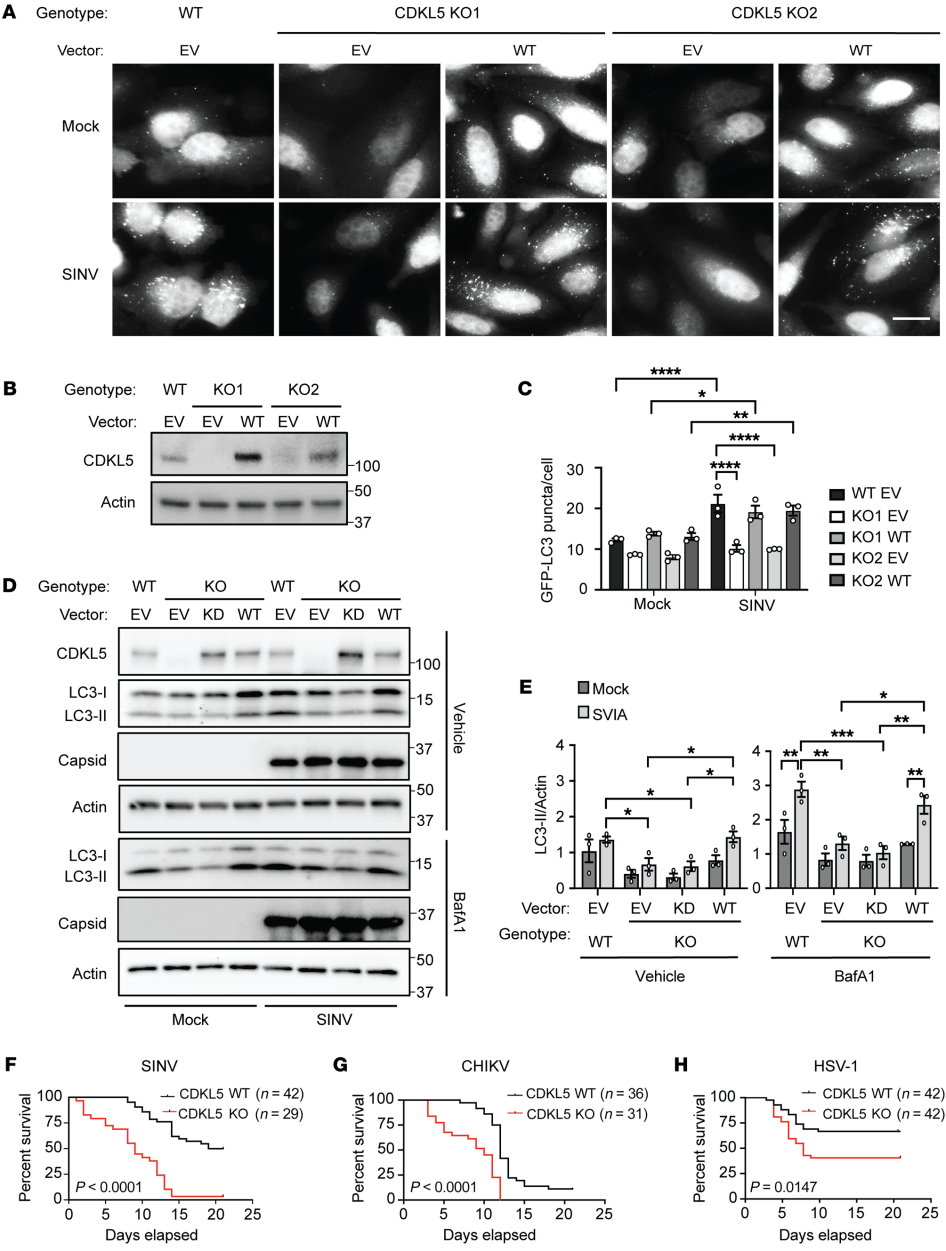
CDKL5 is necessary for SINV–induced autophagy and host antiviral response to diverse viruses. WT CDKL5/GFP-LC3 and 2 CDKL5-KO/GFP-LC3 HeLa clones (KO1 and KO2) were reconstituted with CDKL5 or empty vector (EV). (**A**) Representative fluorescence micrographs of GFP-LC3 puncta (autophagosomes) in mock-infected cells or cells infected with WT SINV at a multiplicity of infection (MOI) of 10 for 6 hours. Scale bar: 20 μm. Data presented here and in Supplemental Figure 1C are from an experiment performed contemporaneously. (**B**) Western blot of CDKL5 expression in WT and CDKL5-KO cells reconstituted with CDKL5 or EV. (**C**) Quantification of GFP-LC3 puncta, with bars representing mean ± SEM of triplicate samples and with at least 100 cells per sample. Statistical analysis was by 1-way ANOVA with Šidák’s correction for multiple comparisons, including the data in Supplemental Figure 1D. Results are representative of 3 independent experiments. (**D**) Western blot of LC3 conversion and (**E**) quantification by densitometry comparing WT and CDKL5-KO cells reconstituted with EV, WT CDKL5, or a kinase dead (KD) CDKL5, infected with SINV (MOI = 10, 6 hours) and treated with either DMSO (vehicle) or BafA1 for 2 hours prior to harvesting cells. Statistical analysis performed by 1-way ANOVA with Dunnett’s test for multiple comparisons. (**F**–**H**) Survival of 7-day-old CDKL5-WT and CDKL5-KO mice infected (**F**) intracerebrally (i.c.) with SINV (1 × 10^3^ PFU) or (**G**) subcutaneously with CHIKV (1 × 10^5^ PFU) and (**H**) 8- to 10-week-old CDKL5-WT and CDKL5-KO mice infected i.c. with HSV-1ΔBBD (5 × 10^4^ PFU). Number of mice per genotype and *P* value (log-rank test) are indicated in **F**–**H**. *P < 0.05; **P < 0.01; ***P < 0.001; ****P < 0.0001.

To investigate the impact of CDKL5 on autophagy during viral infection, we generated 2 *CDKL5*-knockout (CDKL5-KO) clones in HeLa cells stably expressing GFP-LC3, a fluorescent autophagosome marker (11) (Figure 1, A and B). SINV infection of parental HeLa (WT)/GFP-LC3 for 6 hours led to a significant induction of GFP-LC3 puncta (Figure 1, A and C). In contrast, compared with WT HeLa/GFP-LC3 cells, the CDKL5-KO HeLa/GFP-LC3 clones had moderately decreased GFP-LC3 puncta under basal conditions and no induction in autophagosome formation after SINV infection (Figure 1, A and C). CDKL5-KO HeLa cells compared with WT cells also had decreased conversion of LC3-I to the lipidated, autophagosome-associated LC3-II after a 6-hour SINV infection, which was rescued by reconstituting CDKL5-KO cells with WT CDKL5 (Figure 1, D and E, and Supplemental Figure 1, A and B; supplemental material available online with this article; https://doi.org/10.1172/JCI168544DS1). Viral capsid protein was detected to denote the presence of SINV infection. We next tested whether the kinase activity of CDKL5 is required for autophagy induction. We complemented the CDKL5-KO HeLa cells with a kinase-dead mutant by introducing a point mutation that targets the ATP-binding site of the catalytic domain (K42R) that is also a clinically described mutation associated with CDKL5 deficiency disorder (12, 13). Unlike complementation of CDKL5-KO HeLa cells with WT CDKL5, the kinase-dead CDKL5^K42R^ mutant (KD CDKL5) failed to rescue the levels of lipidated LC3-II at baseline or after SINV infection (Figure 1, D and E). Notably, treating cells with bafilomycin A1 (BafA1), a lysosomal acidification and autophagic flux inhibitor (11), caused an increase in LC3-II levels at baseline and after SINV infection in all genotypic backgrounds, indicating that autophagic flux was maintained in CDKL5-deficient cells (Figure 1, D and E). These data indicate that CDKL5, in a kinase activity–dependent manner, is required in HeLa cells for SINV-induced autophagy.

### CDKL5 is required for host defense against diverse viruses.

As autophagy is critical for host antiviral response and survival during infection with several viruses, we investigated whether CDKL5 is necessary for mouse survival after infection with viruses that are targeted for autophagic degradation. We used an intracranial infection model of SINV and herpes strain HSV-1ΔBBD, an enveloped DNA virus engineered to express a deletion mutant of the neurovirulence protein ICP34.5 lacking the Beclin 1–binding domain (ΔBBD). Binding to Beclin 1 through the BBD inhibits autophagy and thus the HSV-1ΔBBD strain fails to inhibit autophagy, which contributes to its reduced virulence in mice (5, 7, 14–16). We also subcutaneously infected mice with CHIKV, an arthropod-borne alphavirus like SINV that causes epidemics of febrile inflammatory arthritis in humans and lethality of neonatal mice (7, 17). Compared with WT mice, CDKL5-KO mice were markedly more susceptible to SINV, HSV-1ΔBBD, and CHIKV infections and succumbed to infection more rapidly than WT mice (Figure 1, F–H), suggesting that CDKL5 has a generalized function in host antiviral defense.

### CDKL5 in neurons regulates virophagy.

**Figure 2:**
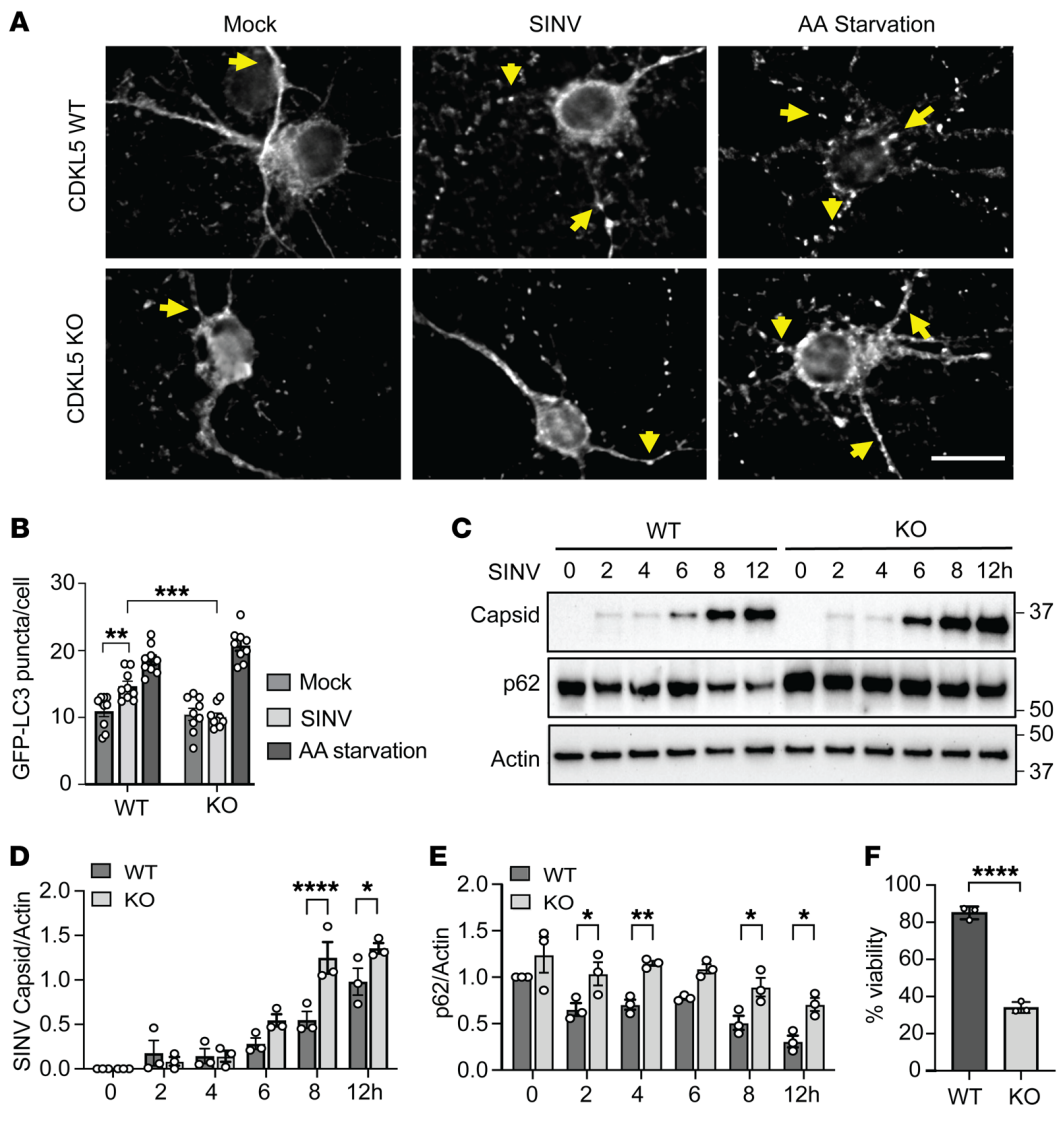
Mouse primary cortical neurons deficient in CDKL5 have defective virophagy but not amino acid starvation–induced autophagy. Primary cortical neurons were isolated from littermate CDKL5-WT/GFP-LC3 and CDKL5-KO/GFP-LC3 transgenic mouse embryos and either maintained in normal media, infected with WT SINV (MOI = 10, 8 hours), or treated with EBSS (amino acid [AA] starvation) medium for 2 hours. (**A**) Representative fluorescence micrographs. Arrows point to representative GFP-LC3 puncta. Scale bar: 20 μm. (**B**) Quantification of GFP-LC3 puncta. Bars are mean ± SEM from triplicate samples (at least 50 neurons per sample counted) from 4 independent embryos per genotype. *P* values were determined by 1-way ANOVA with Dunnett’s test for multiple comparisons. (**C**–**E**) Western blot of capsid accumulation and p62 degradation detected using anti–SINV capsid and anti-p62 antibodies in CDKL5-WT and CDKL5-KO cortical neurons infected with WT SINV (MOI = 10, time points as indicated), with quantification of (**D**) SINV capsid/actin and (**E**) p62/actin ratios from 3 independent experiments. Bars represent mean ± SEM. Statistical analysis performed by 1-way ANOVA test with Šidák’s multiple-comparison test. (**F**) CDKL5-WT and CDKL5-KO cortical neurons were infected with WT SINV (MOI = 10) for 12 hours and cell viability determined by CellTiter-Glo assay (see Supplemental Methods). Bars on graph represent mean ± SEM from 3 independent experiments. *P* values were determined by unpaired, 2-tailed *t* test. *P < 0.05; **P < 0.01; ***P < 0.001; ****P < 0.0001.

Because neurons robustly express CDKL5 (18) and serve as the physiologically relevant cell type for SINV infection, we investigated the autophagic function of CDKL5 in mouse primary cortical neurons isolated from CDKL5-WT/GFP-LC3 and CDKL5-KO/GFP-LC3 transgenic mouse embryos. While the number of GFP-LC3 puncta increased after SINV infection in CDKL5-WT neurons, indicating activation of autophagy, GFP-LC3 puncta were unchanged in infected CDKL5-KO neurons (Figure 2, A and B). Notably, under basal conditions the number of GFP-LC3 puncta in primary cortical neurons was the same between the genotypes (Figure 2, A and B), suggesting a cell-type-specific difference in the regulation of basal autophagy as compared with the modest decrease in GFP-LC3 puncta in CDKL5-KO HeLa cells (Figure 1). Furthermore, absence of CDKL5 in cortical neurons and HeLa cells did not impact autophagy induction by either amino acid starvation or mTOR inhibition based on GFP-LC3 puncta formation and levels of endogenous LC3-II (Figure 2, A and B, and Supplemental Figure 1, C–E). Taken together, these data suggest that CDKL5 regulates SINV-induced autophagy, but not autophagy induced by amino acid starvation in HeLa cells and neurons or mTOR inhibition in HeLa cells.

### CDKL5 controls viral protein clearance and cell survival.

An important function of virophagy is to clear intracellular viral antigens (4). We next tested the consequence of CDKL5 deficiency in primary cortical neurons on levels of SINV capsid protein, a viral structural protein that is generated in excess to favor the encapsulation of viral RNA into nucleocapsids (19). We found CDKL5-deficient cortical neurons had progressively greater accumulation of capsid when compared with WT neurons during SINV infection (Figure 2, C and D). We also monitored the level of p62, a well-established selective autophagy receptor and autophagosome substrate (4), during SINV infection and found that p62 levels decreased over time in WT neurons, whereas CDKL5-KO neurons maintained a higher level of p62 after infection, further suggesting a defect in autophagy in the absence of CDKL5 (Figure 2, C and E). As further evidence of a neuronal cell virophagy defect in the absence of CDKL5, we infected WT and CDKL5-KO cortical neurons expressing GFP-LC3 with SINV for 8 hours and conducted immunofluorescence analysis to determine the colocalization between GFP-LC3 and viral capsid. WT neurons compared with CDKL5-KO neurons had significantly higher numbers of colocalized GFP-LC3 and capsid puncta, suggesting impaired virophagy in the KO neurons (Supplemental Figure 2, A and B). Viral protein clearance via autophagy plays an additional cytoprotective role during viral infection by mitigating the toxicity of viral protein aggregates that accumulate in the cell (1). To determine the impact of CDKL5 on cell survival during viral infection, we infected WT and CDKL5-KO cortical neurons with SINV for 12 hours. Loss of CDKL5 markedly decreased cell viability after SINV infection compared with WT cells, suggesting that CDKL5 promotes cell survival during SINV infection (Figure 2F).

**Figure 3:**
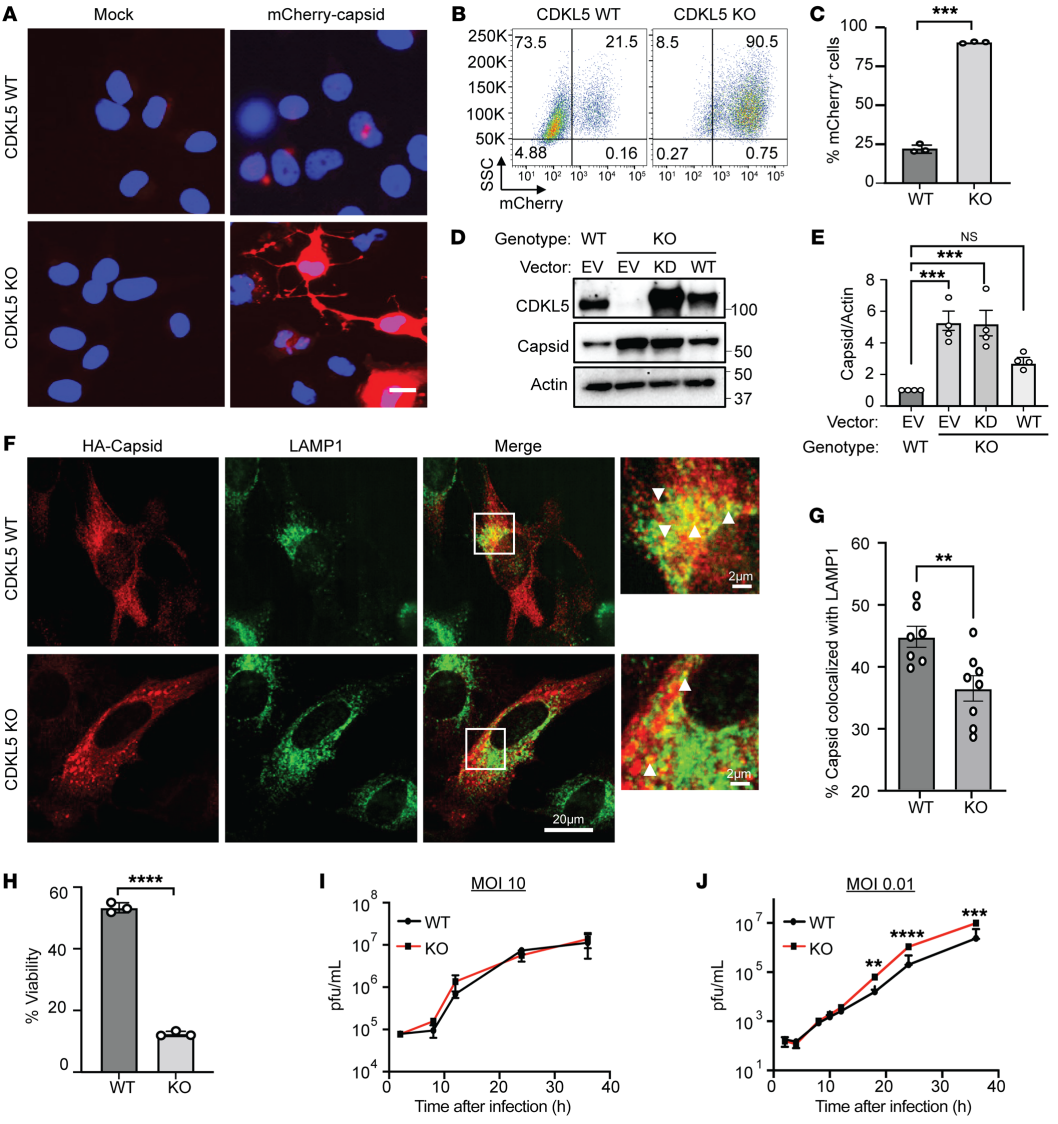
Loss of CDKL5 impairs viral antigen clearance and promotes virus-induced cell death. (**A**–**C**) WT and CDKL5-KO HeLa cells were mock infected or infected with SINV/mCherry-capsid (MOI = 10) for 24 hours. (**A**) Representative fluorescence micrographs of mCherry-capsid. Scale bar: 20 μm. (**B**) Representative flow cytometry plots of mCherry-capsid–positive cells and (**C**) quantification of 3 biological replicates. Bars represent mean ± SEM. *P* values were determined by unpaired, 2-tailed *t* test. (**D** and **E**) WT and CDKL5-KO HeLa cells reconstituted with EV, KD, or WT CDKL5 were infected with SINV/mCherry-capsid (MOI = 10, 24 hours) and (**D**) mCherry-capsid detected by Western blot using an anti–SINV capsid antibody. (**E**) Quantification of capsid/actin ratio from 4 independent experiments. *P* values determined by 1-way ANOVA with Dunnett’s test for multiple comparisons. (**F** and **G**) WT and CDKL5-KO HeLa cells infected with SINV/3×HA-capsid (MOI = 10, 8 hours) and stained with antibodies against LAMP1 and HA for capsid detection. (**F**) Representative immunofluorescence micrographs with (**G**) quantification of colocalization. Bars are mean ± SEM of percentage capsid^+^ puncta colocalized with LAMPI in 8 images (>50 cells). Arrowheads denote examples of capsid^+^/LAMP1^+^ puncta. *P* values were determined by unpaired, 2-tailed *t* test. (**H**) WT and CDKL5-KO HeLa cells were infected with SINV/mCherry-capsid (MOI = 10, 24 hours) and cell viability determined by CellTiter-Glo assay (see Supplemental Methods). Bars on graph represent mean ± SEM from 3 independent experiments. *P* values were determined by unpaired, 2-tailed *t* test. (**I**) High-MOI (MOI = 10) and (**J**) low-MOI multistep growth curve analysis (MOI = 0.01) of WT and CDKL5-KO HeLa cells infected with SINV/mCherry-capsid. Progeny viruses were quantified by plaque assays. Bars represent mean ± SD of 3 experimental replicates and *P* values were determined by 2-way ANOVA with Šidák’s multiple-comparison test. **P < 0.01; ***P < 0.001; ****P < 0.0001.

To further investigate whether HeLa cells also require CDKL5 to mitigate capsid accumulation, we infected cells with an attenuated recombinant SINV expressing an mCherry-capsid fusion protein at a high MOI of 10 for 24 hours to allow for greater measurable differences between CDKL5-WT and -KO cells. We visualized capsid via fluorescence microscopy (Figure 3A) and quantified mCherry-capsid–positive cells through flow cytometry (Figure 3, B and C). Loss of CDKL5 resulted in robust intracellular accumulation of mCherry-capsid protein that saturated the cell with large aggregates (Figure 3, A–C), suggesting a defect in virophagy. Reconstitution of CDKL5-KO HeLa cells with WT CDKL5 significantly decreased capsid levels, whereas reconstitution with the KD CDKL5 mutant failed to rescue the capsid accumulation phenotype observed in CDKL5-deficient cells (Figure 3, D and E). Thus, we conclude that CDKL5 through its kinase activity mitigates capsid accumulation during SINV infection.

Complete autophagic degradation of cargo requires maturing autophagosomes to ultimately fuse with lysosomes to form LAMP1^+^ autolysosomes where cargo is degraded (11). To further establish whether CDKL5 impacted the targeting of capsid proteins to LAMP1^+^ autolysosomes, we performed immunofluorescence microscopy and analysis of capsid and LAMP1 colocalization. We infected HeLa cells with a recombinant SINV expressing 3×HA-tagged capsid for 8 hours and detected capsid through anti-HA antibodies. We chose this early time point to avoid the saturation of CDKL5-KO cells with capsid that occurs at later time points. We observed that loss of CDKL5 significantly diminished the colocalization of capsid and LAMP1 (Figure 3, F and G), further indicating defective virophagy. To determine whether CDKL5 had a generalized role in virophagy, we infected CDKL5-WT and -KO HeLa cells with HSV-1ΔBBD and visualized virions captured within double-lipid-membrane autophagic vacuoles using electron microscopy (EM). We chose to use HSV-1 because its intracellular virus assembly and virion structure in relation to autophagic vacuoles are well studied and easy to visualize by EM (15, 16). HSV-1 undergoes nucleocapsid assembly in the nucleus, but these nucleocapsids can be captured by autophagosomes upon reentry into the cytosol prior to egress. In CDKL5-KO cells, nucleocapsids observably tended to be both inside and outside autophagic vesicles, while in WT cells, the virions primarily resided within autophagic vacuoles (Supplemental Figure 3).

We next investigated whether CDKL5 had a cytoprotective role in SINV-infected HeLa cells like in cortical neurons. We infected WT and CDKL5-KO HeLa cells with SINV/mCherry-capsid for 24 hours and found significantly increased cell death in the CDKL5-deficient cells (Figure 3H), confirming the importance of CDKL5 in affording protection from virus-induced cell death.

Given the increased susceptibility of CDKL5-KO HeLa cells to SINV infection, we tested whether CDKL5 impacted overall viral replication in HeLa cells using viral growth assays that test the production of infective progeny virions. Prior studies demonstrated that although autophagy is essential for cell survival during viral infection, impaired autophagic clearance of viral proteins does not necessarily lead to markedly increased viral replication (5, 7, 15, 16). Like prior studies, despite our observation of robust capsid accumulation in CDKL5-KO HeLa cells compared with WT at an MOI of 10 (Figure 3, A–C), we observed no difference in replication of SINV/mCherry-capsid virus between WT and CDLK5-KO cells when we performed a high-MOI growth curve analysis (MOI = 10; Figure 3I). However, we noted a slight increase in viral replication in CDKL5-KO cells in a low-MOI (MOI = 0.01) multicycle growth curve experiment (Figure 3J), suggesting that CDKL5 plays a modest role in mitigating viral replication.

### CDKL5 regulates viral capsid clearance independently of viral replication.

**Figure 4:**
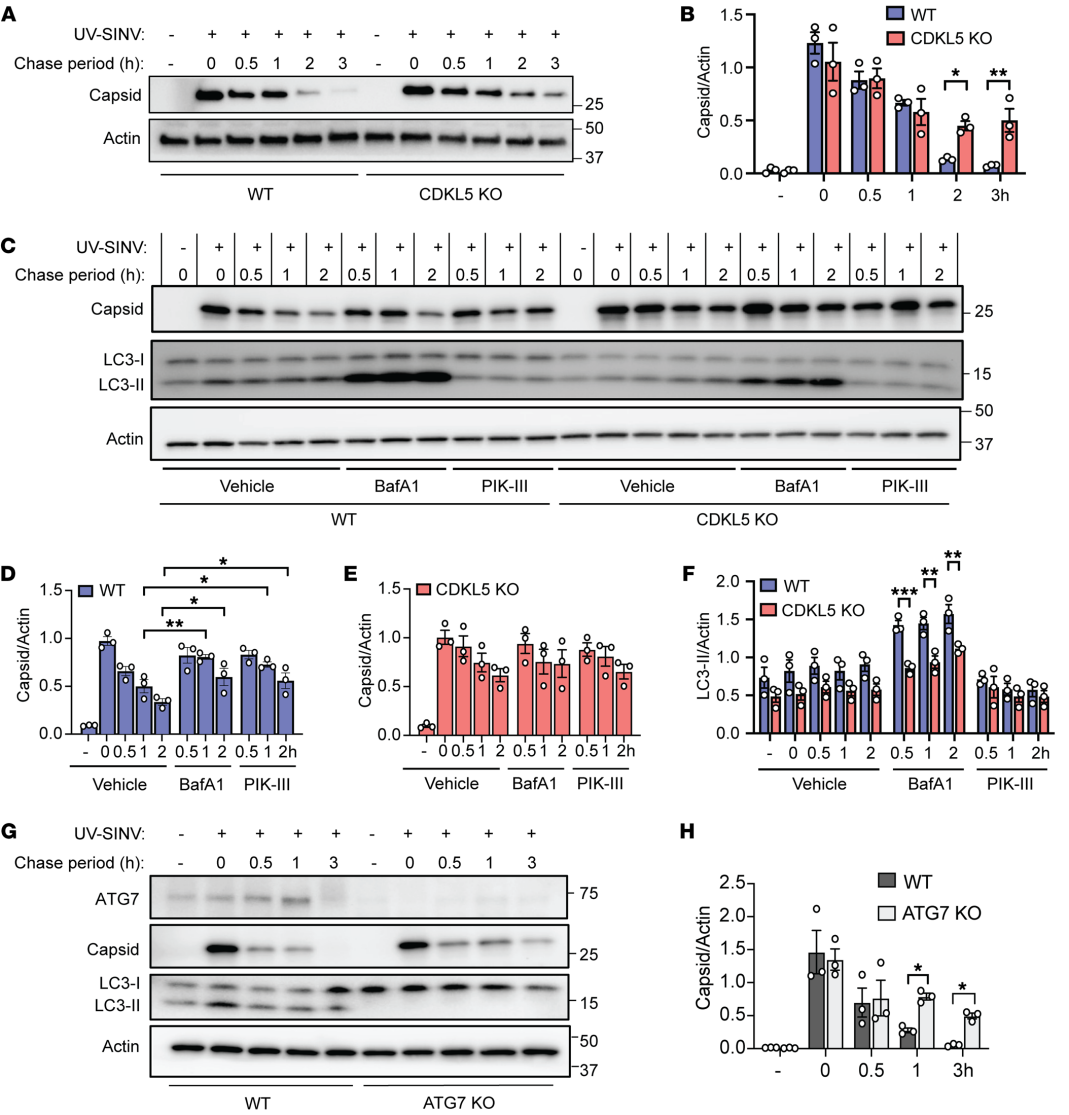
Loss of CDKL5 delays autophagy-mediated clearance of capsid in cells pulsed with nonreplicating SINV. (**A** and **B**) WT and CDKL5-KO HeLa cells exposed to UV-inactivated WT SINV (equivalent to MOI of 500) for 1 hour, washed, and lysates harvested at the indicated time points. (**A**) Western blot of capsid degradation and (**B**) quantification of capsid/actin ratio by densitometry. Bars are mean ± SEM from 3 independent experiments. *P* values were determined by 1-way ANOVA test with Šidák’s multiple-comparison test. (**C**–**F**) WT and CDKL5-KO HeLa cells exposed to UV-inactivated SINV for 1 hour, treated with DMSO (vehicle), BafA1 (100 μM), or PIK-III (5 μM) and cell lysates analyzed by (**C**) Western blot for capsid degradation and LC3 conversion. (**D**) Quantification of capsid/actin ratio in WT cells. (**E**) Quantification of capsid/actin ratio in CDKL5-KO cells. (**F**) Quantification of LC3-II normalized to actin. (**D**–**F**) Bars are mean ± SEM from 3 independent experiments. *P* values in **D** and **E** were determined by 1-way ANOVA with Dunnett’s test for multiple comparisons and in **F** by 2-way ANOVA with Tukey’s multiple-comparison test. (**G** and **H**) WT and ATG7-KO cells were exposed to UV-inactivated SINV for 1 hour, washed, and then lysates harvested at indicated time points. (**G**) Western blot of ATG7, capsid, and LC3-I/LC3-II and (**H**) quantification of capsid/actin ratio by densitometry. Bars are mean ± SEM from 3 independent experiments. *P* values were determined by 1-way ANOVA with Šidák’s multiple-comparison test. *P < 0.05; **P < 0.01; ***P < 0.001.

Since CDKL5 may restrict SINV capsid accumulation through several mechanisms that affect the synthesis and clearance of capsid, we tested the impact of CDKL5 deficiency on viral capsid clearance independent of viral replication. To that end, we used a UV-inactivated SINV capable of cellular uptake but not replication. To detect autophagy induction by UV-inactivated SINV, we pulsed WT and CDKL5-KO HeLa cells with UV-inactivated SINV equivalent to an MOI of 500 and, after removing extracellular virus, quantified capsid clearance over time. Although both WT and CDKL5-KO cells internalized UV-inactivated SINV equally, capsid was cleared by 2–3 hours in WT cells but persisted in CDKL5-KO cells (Figure 4, A and B). We further investigated the role of autophagy in capsid clearance by treating WT and CDKL5-KO cells pulsed with UV-inactivated SINV with either BafA1 or PIK-III, a class III phosphatidylinositol-3 kinase (PI3KC3, also known as VPS34) inhibitor, which inhibits PI3KC3 C1 and C2 complexes essential for autophagosome membrane nucleation and lysosome fusion (20). Whereas treatment of WT cells significantly stabilized capsid levels after 1 hour of the chase period compared with vehicle, treatment of CDKL5-KO cells with either inhibitor had no significant effect on capsid accumulation (Figure 4, C–E). Similarly, WT cells pulsed with UV-inactivated virus had a greater accumulation of lipidated LC3 in the presence of BafA1, whereas PIK-III–treated cells did not accumulate lipidated LC3 (Figure 4, C and F). To confirm that capsid clearance is autophagy dependent, we also determined the rate of capsid clearance by autophagy-deficient HeLa ATG7-KO cells (Figure 4G). Absence of ATG7 resulted in delayed capsid clearance compared with WT cells (Figure 4, G and H), indicating that autophagy plays a role in capsid clearance. Notably, SINV capsid could still be cleared by ATG7-KO and CDKL5-KO HeLa cells, albeit more slowly, suggesting that other mechanisms can reduce SINV capsid accumulation in the absence of ATG7 and CDKL5.

We next investigated whether the ubiquitin proteasome system (UPS) facilitates capsid degradation in addition to autophagy by treating cells with epoxomicin, an irreversible proteosome inhibitor. We found that epoxomicin partially rescued capsid degradation in WT and CDKL5-KO HeLa cells, suggesting that capsid degradation requires both autophagy and UPS (Supplemental Figure 4). Taken together, these findings demonstrate that CDKL5 mediates the efficient clearance of capsid from UV-inactivated SINV in an autophagy-dependent manner.

### CDKL5 promotes association of viral capsid with p62.

**Figure 5:**
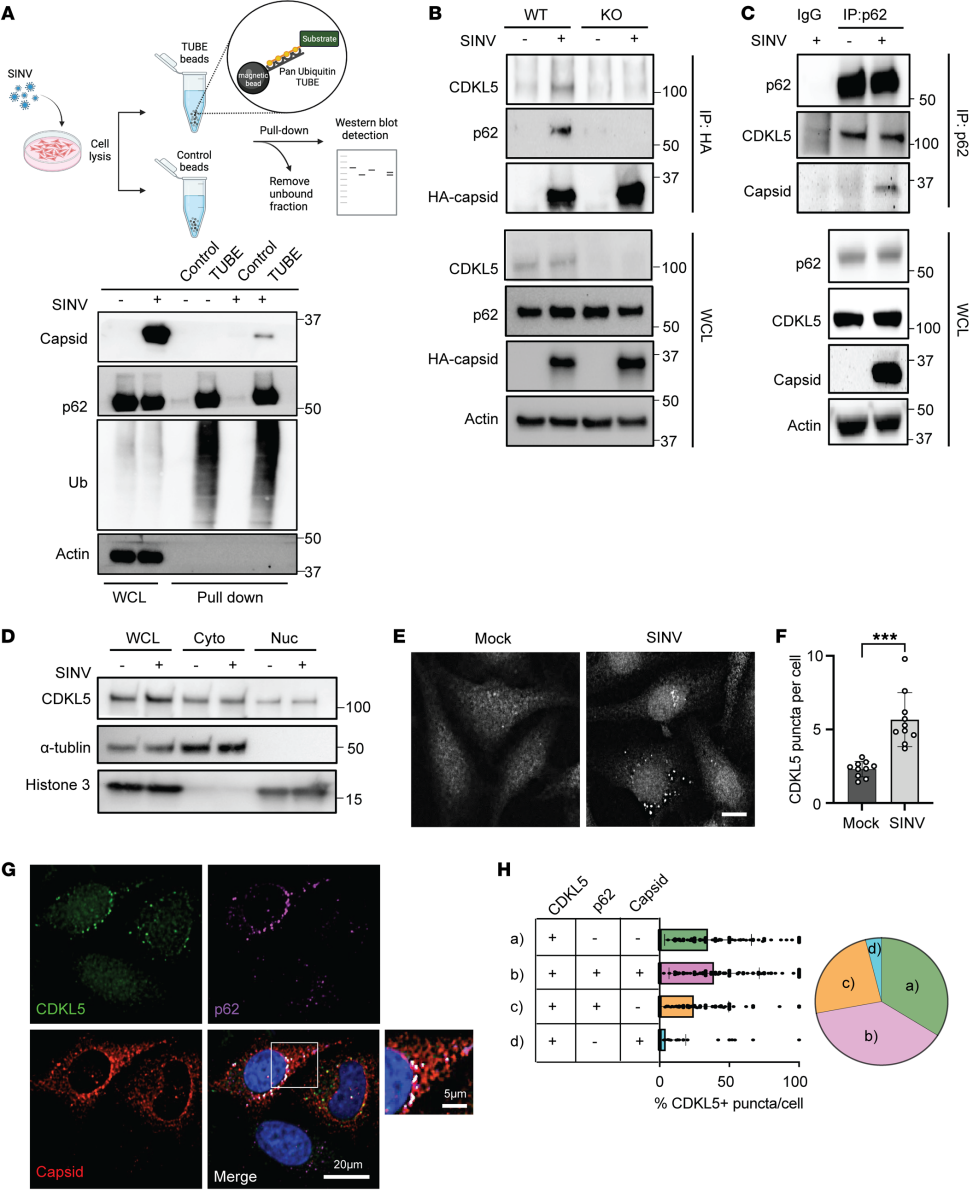
CDKL5 is necessary for the interaction of SINV capsid with p62. (**A**) A pan-ubiquitin TUBE conjugated to magnetic beads or unconjugated magnetic beads (control) were used to pull down ubiquitinated proteins from uninfected or SINV-infected WT HeLa cells. Capsid, ubiquitin, and p62 detected on Western blot. WCL, whole-cell lysate. (**B**) Immunoblot of endogenous CDKL5, p62, and HA-capsid after HA-capsid coimmunoprecipitation from CDKL5-WT and CDKL5-KO HeLa cells infected with SINV/HA-capsid (MOI = 10, 7 hours). Results are representative of 3 independent experiments. (**C**) Immunoprecipitation of endogenous p62 in CDKL5-KO HeLa cells expressing WT CDKL5–3×FLAG and infected with WT SINV for 8 hours. CDKL5 was detected with anti-FLAG antibody and capsid with anti–SINV capsid antibody. Results are representative of 3 independent experiments. (**D**) Nuclear/cytoplasmic fractionations were performed using 0.1% NP-40 lysis buffer of WT HeLa cells either mock infected or infected with WT SINV for 8 hours. Endogenous CDKL5 was detected on immunoblots with histone 3 used as a marker for purity of the nuclear extracts and α-tubulin for the cytoplasmic extracts. (**E**) CDKL5 detected and imaged in WT HeLa cells either mock infected or infected with WT SINV for 8 hours. Scale bar: 10 μm. (**F**) Quantification of CDKL5 puncta per cell within each image. Ten total images quantified with more than 300 cells. *P* values were determined by unpaired, 2-tailed *t* test. ***P < 0.001. (**G**) WT HeLa cells were infected with WT SINV (MOI = 10) for 8 hours then analyzed by immunofluorescence microscopy. (**H**) Bar graph representative of the percentage of CDKL5 puncta per cell that are positive for p62, capsid, both, or only CDKL5. Pie chart represents the overall distribution of CDKL5 puncta colocalizing with p62, capsid, both, or neither.

p62 generally recognizes and recruits ubiquitinated cargos to autophagosomes for degradation (21). We predicted that SINV capsid, which has been shown to interact with p62 (5), undergoes ubiquitination. To test whether SINV capsid is ubiquitinated, we affinity purified ubiquitinated proteins from cellular lysates using a pan-ubiquitin tandem ubiquitin-binding entity (TUBE) followed by immunoblotting to identify capsid. We found that a small but detectable fraction of capsid undergoes ubiquitination, while p62, known to undergo ubiquitination, was heavily ubiquitinated and served as a positive control (Figure 5A). Because CDKL5-deficient cells demonstrated defective autophagy induction and delayed capsid and p62 clearance, we next sought to determine whether CDKL5 impacts the interaction of SINV capsid with p62 to initiate virophagy. We infected WT and CDKL5-KO HeLa cells and primary cortical neurons with SINV/mCherry-capsid and performed immunofluorescence colocalization studies at an early time point before cells lacking CDKL5 became saturated with capsid (Supplemental Figure 5). We observed a greater number of colocalized mCherry-capsid–p62 puncta in WT compared with CDKL5-KO cells (Supplemental Figure 5, A and B), suggesting that CDKL5 regulated the p62-capsid interaction.

To further characterize the association between p62 with SINV capsid, we infected WT and CDKL5-KO HeLa cells with recombinant SINV expressing HA-tagged capsid and performed coimmunoprecipitation of p62 after HA pull-down. As expected, endogenous p62 in WT cells coimmunoprecipitated with HA-capsid (Figure 5B). Surprisingly, CDKL5 also coimmunoprecipitated with HA-capsid (Figure 5B). However, in the absence of CDKL5, we did not detect p62 after HA-capsid immunoprecipitation despite higher levels of capsid in the cell lysates (Figure 5B) and similar p62 transcript levels (Supplemental Figure 6A). In addition, baseline levels of the core selective autophagy receptors NBR1, p62, NDP52, OPTN, and TAX1BP1 were similar when comparing WT and CDKL5-KO cells (Supplemental Figure 6B). To validate the interaction between p62 and capsid, we immunoprecipitated endogenous p62 in uninfected or SINV-infected WT HeLa cells. Western blot analysis of the immunoprecipitates revealed the presence of capsid, confirming the p62-capsid interaction. Additionally, we observed CDKL5 immunoprecipitating with p62 from both the uninfected and infected cell lysates, suggesting that CDKL5 can associate with p62 even in the absence of infection (Figure 5C). Taken together, these data suggest that CDKL5 interacts with p62 and facilitates the interaction of p62 with SINV capsid.

We next examined whether CDKL5 localization changes with SINV infection to associate with p62 and capsid structures in the cell. As CDKL5 has both nuclear localization and export signals (22), we first fractionated cellular lysates of uninfected and infected cells to nuclear and cytosolic fractions and determined that a greater portion of CDKL5 resides in the cytosolic fraction (Figure 5D). The distribution of CDKL5 within these compartments did not change with infection (Figure 5D). However, using immunofluorescence, we found that cytosolic CDKL5 changed from being diffuse in uninfected HeLa cells to coalescing into punctate structures after infection with SINV (Figure 5, E and F). The greatest proportion of CDKL5 puncta (39%) colocalized with both capsid and p62, 35% with p62 alone and 25% did not colocalize with either (Figure 5, G and H). Notably, only a small portion (4%) of CDKL5 puncta colocalized with capsid in the absence of p62 (Figure 5, G and H), suggesting that p62 may function as an important link for CDKL5-mediated autophagic degradation of capsid.

**Figure 6:**
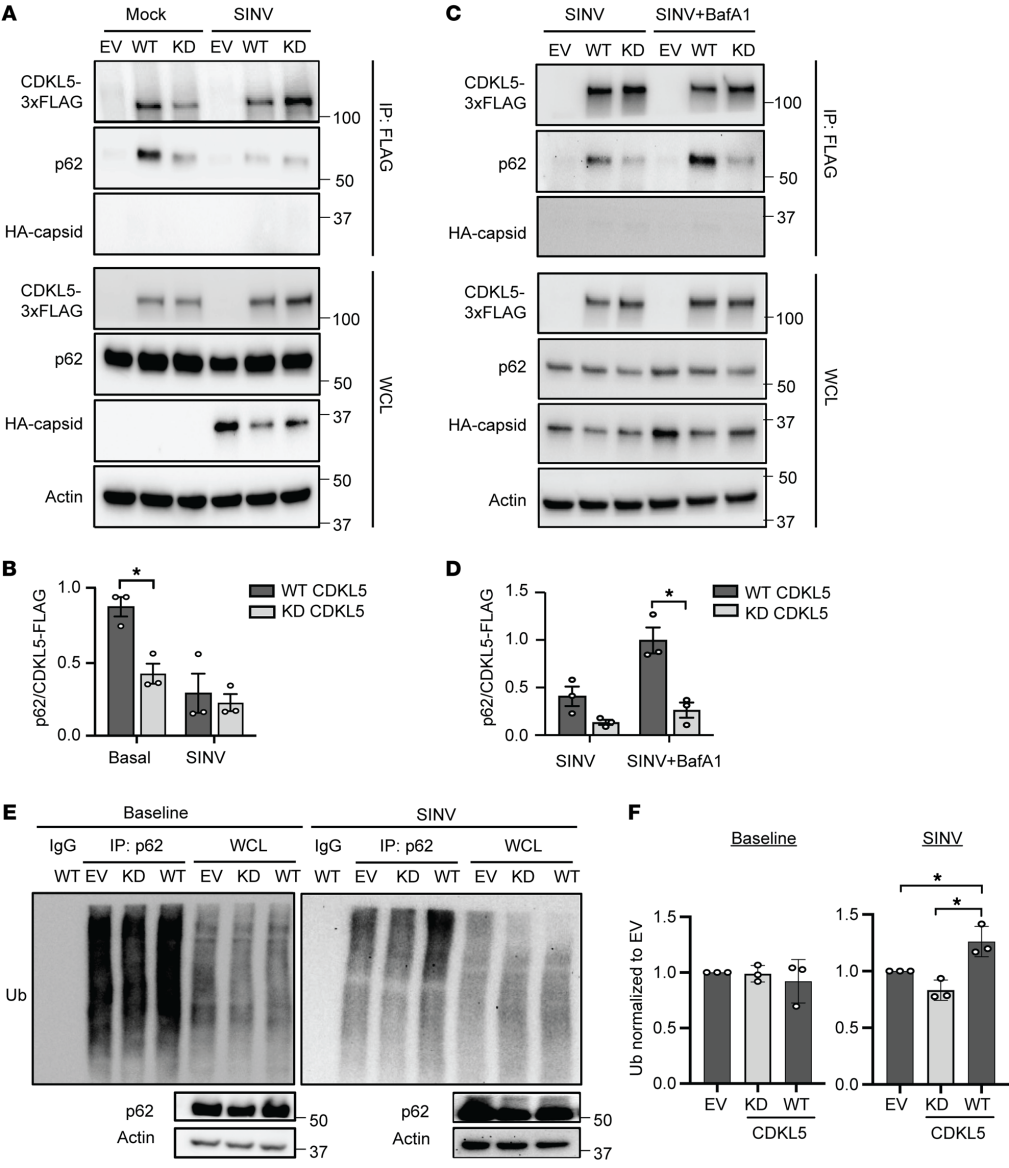
CDKL5 kinase activity facilitates p62 degradation and binding to ubiquitinated cargo during SINV infection. (**A** and **B**) CDKL5-KO HeLa cells reconstituted with EV, WT, or KD CDKL5*–*3×FLAG were infected with SINV/HA-capsid (MOI = 10, 8 hours). (**A**) Immunoblot with anti-HA, anti-p62, and anti-FLAG antibodies after CDKL5–3×FLAG immunoprecipitation with anti-FLAG antibody. (**B**) Quantification of p62 that coimmunoprecipitated with WT or KD CDKL5-FLAG normalized to FLAG. Bars are mean ± SEM from 3 independent experiments. (**C** and **D**) CDKL5-KO HeLa cells reconstituted and infected with SINV as in **A** were treated with either vehicle (DMSO) or BafA1 (100 μM) for 2 hours, followed by immunoblotting and quantification of p62 coimmunoprecipitation with CDKL5 (**B**). Bars are mean ± SEM from 3 independent experiments. (**E** and **F**) CDKL5-KO HeLa cells reconstituted as in **A** were infected with WT SINV for 8 hours (MOI = 10) and lysates from uninfected (baseline) or infected cells subjected to endogenous p62 immunoprecipitation. Ubiquitin (Ub) was detected via a pan-ubiquitin antibody. (**E**) Representative blot and (**F**) quantification of ubiquitin normalized to the level of EV. Bars are mean ± SEM from 3 independent experiments. All *P* values were determined by 1-way ANOVA with Šidák’s multiple-comparison test. *P < 0.05.

Because CDKL5 kinase activity was necessary for the autophagic clearance of capsid, we further investigated whether CDKL5 kinase activity impacts the interaction between CDKL5, p62, and capsid. We expressed WT CDKL5-FLAG and KD CDKL5-FLAG in CDKL5-KO HeLa cells and performed anti-FLAG immunoprecipitation in mock-infected or SINV/HA-capsid–infected cells. In alignment with the immunofluorescence findings in Figure 5G, neither WT nor KD CDKL5-FLAG immunoprecipitation resulted in detectable HA-capsid coimmunoprecipitation (Figure 6, A and B). In uninfected cells, we detected p62 after immunoprecipitation of WT CDKL5-FLAG and to a lesser extent after immunoprecipitation with the KD CDKL5 (Figure 6, A and B), supporting the finding that CDKL5 interacts with p62 even in the absence of infection. Notably, when cells were infected with SINV, the coimmunoprecipitation of p62 by WT CDKL5-FLAG was greatly diminished (Figure 6, A and B). We hypothesized that since p62 undergoes autophagic degradation, infecting cells expressing WT CDKL5-FLAG but not KD CDKL5-FLAG with SINV stimulates autophagic activity, resulting in decreased p62 bound to CDKL5. As predicted, blocking autophagic flux and thus p62 degradation with BafA1 restored the p62 that coimmunoprecipitated with WT CDKL5-FLAG, but not p62 that coimmunoprecipitated with KD CDKL5-FLAG (Figure 6, C and D). We next explored whether CDKL5 kinase activity generally impacts the interaction between p62 and ubiquitinated substrates during SINV infection. We immunoprecipitated endogenous p62 from uninfected or SINV-infected CDKL5-KO HeLa cells expressing an empty vector (EV), WT CDKL5-FLAG, or KD CDKL5-FLAG (Figure 6E). In all 3 HeLa lines, the levels of ubiquitinated substrates pulled down by p62 at baseline appeared the same (Figure 6, E and F). In contrast, after an 8-hour infection with SINV, p62 from cells expressing WT CDKL5 coimmunoprecipitated a greater amount of ubiquitinated substrate compared with infected cells expressing EV or KD CDKL5-FLAG (Figure 6, E and F). Taken together, our data demonstrate that CDKL5 interacts with p62, and the kinase activity of CDKL5 is necessary for p62’s interaction with ubiquitinated substrates and autophagic degradation of p62 during viral infection.

### CDKL5 phosphorylates p62 to promote binding to capsid.

**Figure 7:**
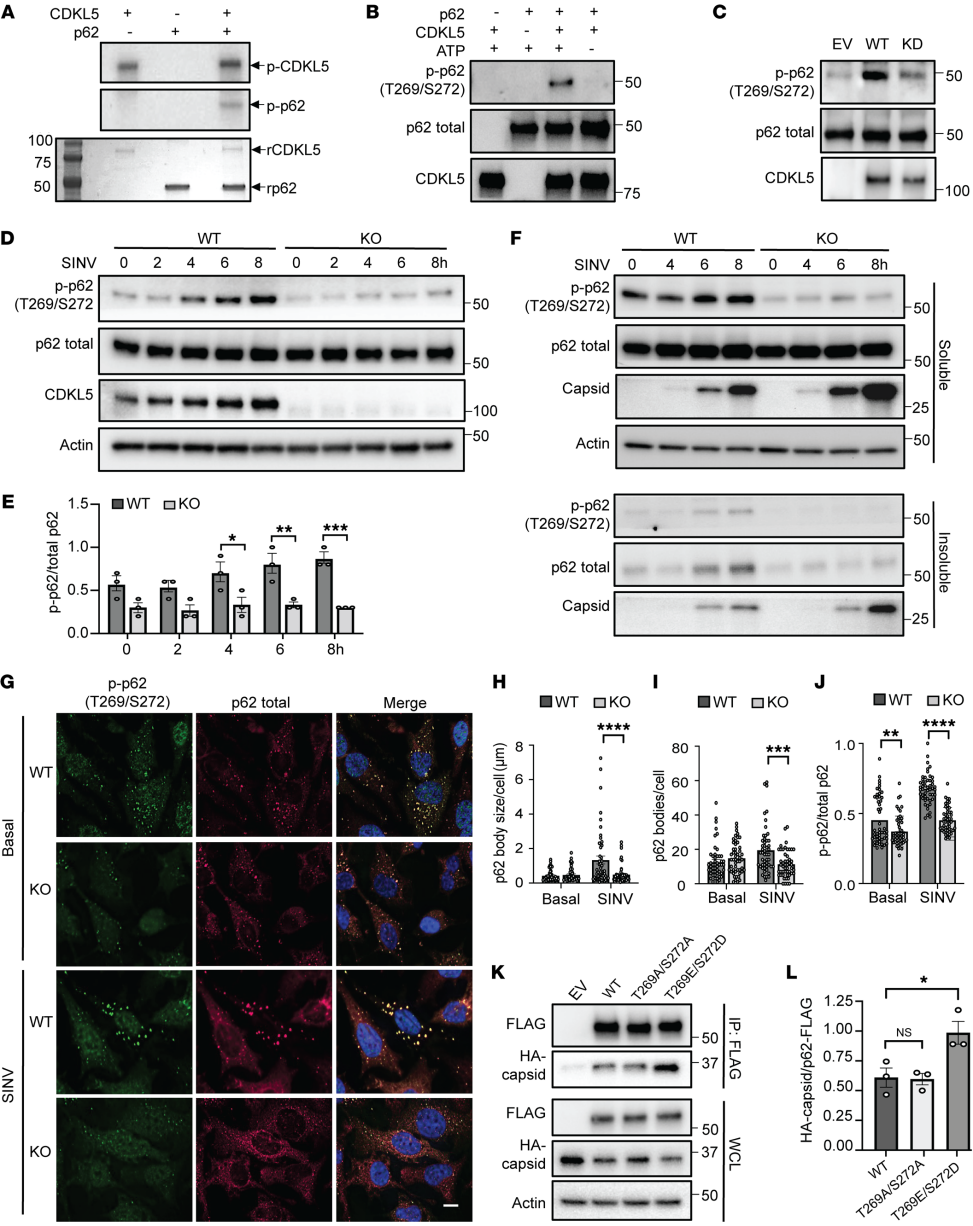
CDKL5 phosphorylates p62 at Thr269/Ser272 to impact binding of p62 to capsid. (**A**) In vitro kinase assay with recombinant CDKL5 (rCDKL5) and rp62 using [γ-^32^P]ATP for a 30-minute reaction. Top: ^32^P autoradiography of p62 and CDKL5 phosphorylation. Bottom: Coomassie Brilliant Blue gel staining. (**B**) In vitro kinase assay with p62 phosphorylation detected using anti-T269/S272 phosphospecific antibody. (**C**) In vitro kinase assay of affinity-purified EV, WT, and KD CDKL5–3×FLAG from HeLa cells and rp62 followed by p62 T269/S272 phosphorylation detection. Blot representative of 3 independent experiments. (**D**) WT and CDKL5-KO HeLa cells infected with SINV/HA-capsid (MOI = 10) were lysed directly in Laemmli buffer. Blot representative of 3 independent experiments. (**E**) Quantification of p-T269/S272 normalized to total p62. Bars are mean ± SEM. (**F**) Fractionation of SINV/HA-capsid–infected WT and CDKL5-KO HeLa cells into RIPA buffer–soluble and –insoluble fractions. Blot is representative of 3 independent experiments. (**G**–**J**) Immunofluorescence microscopy of WT and CDKL5-KO HeLa cells infected with WT SINV (MOI = 10, 8 hours). (**G**) Representative fluorescence micrographs. Analysis of (**H**) size, (**I**) count, and (**J**) ratio of p-T269/S272 p62 to total p62 bodies per cell with 50 cells per condition. Bars are mean ± SEM. Scale bar: 10 μm. (**K** and **L**) FLAG immunoprecipitation of SINV/HA-capsid virus–infected CDKL5-KO HeLa cells expressing EV, WT p62, alanine-substituted p62^T269A/S272A^, or phosphomimetic p62^T269E/S272D^-3×FLAG. (**K**) Representative blot. (**L**) Densitometry analysis of capsid coimmunoprecipitating with p62-FLAG normalized to FLAG. Bars are mean ± SEM of 3 experiments. *P* values were determined by 1-way ANOVA with Šidák’s multiple-comparison test (**E** and **H**–**J**) or 1-way ANOVA with Dunnett’s multiple-comparison test (**L**). *P < 0.05; **P < 0.01; ***P < 0.001; ****P < 0.0001.

Since the interaction between CDKL5 and p62 required CDKL5 kinase activity, we hypothesized that CDKL5 directly phosphorylates p62. To test this, we performed in vitro phosphorylation assays using recombinant CDKL5 and p62 proteins (Supplemental Figure 7A). As expected (23), in the absence of p62, CDKL5 underwent autophosphorylation, thus serving as a positive control for its catalytic activity (Figure 7A). While p62 alone did not autophosphorylate, combining CDKL5 with p62 resulted in phosphorylation of p62 (Figure 7A). An in vitro phosphorylation time course showed that CDKL5 autophosphorylation, which leads to autoactivation of the kinase, preceded the phosphorylation of p62, thus providing further evidence that p62 is a substrate of CDKL5 (Supplemental Figure 7B).

We next sought to identify a site on p62 phosphorylated by CDKL5. p62 has multiple established phosphorylation sites (21, 24), and antibodies are commercially available for the most common sites. We initially hypothesized that CDKL5 phosphorylates p62 at S403 because phosphorylation of this site increases the binding affinity of p62 to ubiquitinated cargo during selective autophagy, including bacterial xenophagy (25–27). To test our hypothesis, we used an in vitro phosphorylation assay to achieve CDKL5 phosphorylation of p62 but found no detectable S403 phosphorylation (Supplemental Figure 7C). In contrast, tank-binding kinase 1 (TBK-1), which is known to phosphorylate p62 at S403 (26, 27), led to detectable S403 phosphorylation (Supplemental Figure 7C).

Since our data demonstrated that the absence of CDKL5 reduced colocalization of p62 with capsid within punctate structures (Supplemental Figure 5), we hypothesized that CDKL5 phosphorylation influences the ability of p62 to cluster around capsid. Notably, during proteotoxic stress caused by proteasome inhibition, phosphorylation of p62 at T269/S272 by p38 MAPK facilitates the formation of p62 aggresomes that sequester misfolded proteins to initiate autophagosome biogenesis (28). However, phosphorylation of these residues has not been described during viral infection. To test whether CDKL5 phosphorylates p62 at T269/S272, we performed in vitro kinase assays and probed with an antibody specific for phosphorylated T269/S272. We detected p-p62 (T269/S272) only in the presence of both CDKL5 and ATP (Figure 7B). As an additional line of evidence, we affinity purified WT CDKL5-FLAG or KD CDKL5-FLAG expressed in CDKL5-KO HeLa cells and tested the ability of WT and KD CDKL5 to phosphorylate recombinant p62 in vitro. We found greater phosphorylation of p62 in the presence of WT CDKL5 when compared with EV-FLAG and KD CDKL5-FLAG (Figure 7C).

To test the impact of SINV infection on CDKL5-dependent T269/S272 phosphorylation of endogenous p62, we infected WT and CDKL5-KO HeLa cells with SINV and assessed p62 T269/S272 phosphorylation over time. WT HeLa cells demonstrated not only higher basal p-T269/S272 p62 compared with CDKL5-KO cells, but also a significantly greater induction of T269/S272 p62 phosphorylation after infection with SINV, which correlated with increasing levels of CDKL5 (Figure 7, D and E; compare time = 0). In fact, CDKL5-KO cells failed to phosphorylate p62 at T269/S272 after infection (Figure 7, D and E). Taken together, these findings demonstrate that CDKL5 phosphorylates p62 at T269/S272 and SINV infection robustly induces CDKL5-dependent T269/S272 p62 phosphorylation.

We next sought to determine whether p62 aggregation was defective in the absence of CDKL5 during SINV infection. Whereas we previously assessed p62 levels in total cell lysates (Figure 7D), to detect p62 aggresome formation we fractionated cell lysates to RIPA-soluble and -insoluble fractions from SINV-infected WT and CDKL5-KO HeLa cells, as nascent p62 aggresomes are predicted to partition into the insoluble fraction (29). While soluble total p62 levels were equal between SINV-infected WT and CDKL5-KO HeLa cells, p62 accumulated to a greater extent in the insoluble fraction isolated from WT than KO cells (Figure 7F). Furthermore, p-p62 T269/S272 was detectable in the insoluble fraction of WT but not KO cells (Figure 7F). Interestingly, CDKL5-KO cells overall had more capsid in both the soluble and insoluble fractions, suggesting that while aggregation of p62 depends on CDKL5, capsid aggregation is independent of CDKL5 (Figure 7F). We further examined the clustering of p62 into large intracellular bodies by immunofluorescence imaging and found that compared with SINV-infected CDKL5-KO cells, WT cells had significantly larger and greater numbers of p62 bodies, with proportionately more p-T269/S272 contained within these bodies (Figure 7, G–J). These findings indicate that CDKL5-deficient cells are defective in the formation and incorporation of p-p62 T269/S272 into p62 aggresomes.

Since the oligomerization of p62 into large bodies functions in cargo recruitment to autophagosomes (30, 31), we predicted that if we overexpressed the phosphomimetic mutant p62^T269E/S272D^ (28) in CDKL5-KO cells, we could rescue p62’s interaction with capsid. To that end, we stably expressed FLAG-tagged versions of WT p62, p62 that cannot be phosphorylated (p62^T269A/S272A^), and the phosphomimetic mutant (p62^T269E/S272D^) in CDKL5-KO HeLa cells. We assessed the interaction between p62 and HA-capsid through coimmunoprecipitation using an anti-FLAG antibody. WT p62 and p62^T269A/S272A^ bound similar levels of capsid (Figure 7, K and L). In contrast, the phosphomimetic mutant p62^T269E/S272D^-FLAG bound significantly more capsid (Figure 7, K and L), suggesting that CDKL5 phosphorylation of these residues facilitates the interaction between p62 and capsid.

To investigate the importance of p62 phosphorylation for the autophagic clearance of SINV capsid, we performed immunofluorescence microscopy to examine the colocalization between capsid and LAMP1, in SINV-infected CDKL5-KO HeLa cells expressing EV, p62^WT^, p62^T269A/S272A^, or p62^T269E/S272D^. Capsid^+^ puncta colocalization with LAMP1 was lowest in cells expressing EV, intermediate in cells expressing p62^WT^ or p62^T269A/S272A^, and the highest with p62^T269E/S272D^, supporting the hypothesis that p62 phosphorylation at T269/S272 facilitates the targeting of SINV capsid to autolysosomes for degradation (Supplemental Figure 8A). To corroborate these findings, we assessed the protein stability of capsid and p62 within the same cell lines through a cycloheximide (CHX) chase assay. Cells were infected with WT SINV for 6 hours, an early time point in which the initial capsid protein level (time 0) between the HeLa strains was similar, followed by a 7-hour chase period after the addition of CHX (Supplemental Figure 8B). Western blot analysis revealed that the degradation of capsid and p62 occurred faster in cells expressing p62^T269E/S272D^ compared with those expressing EV, p62^WT^, or p62^T269A/S272A^. These data suggested that the phosphomimetic p62 augmented the degradative capabilities of the CDKL5-KO cells (Supplemental Figure 8, B and C). We additionally generated 2 autophagy-defective mutants of p62 to serve as negative controls: p62^LIR^ ^mut^ that is incapable of binding to LC3 and p62^ΔUBA^ that lacks the ubiquitin-binding domain needed for cargo binding. We infected CDKL5-KO HeLa cells expressing the p62 mutants with SINV for 8 hours and assessed for differences in capsid accumulation by Western blot analysis. We found p62^LIR^ ^mut^ and p62^ΔUBA^ had increased capsid levels like cells containing EV (Supplemental Figure 8D). Conversely, cells expressing p62^WT^ and p62^T269E/S272D^ had decreased capsid accumulation, with the phosphomimetic mutant accumulating the least capsid (Supplemental Figure 8D). Overall, these data suggest that CDKL5-mediated phosphorylation of p62 at T269/S272 enables p62 to facilitate the autophagic degradation of capsid.

### Phosphorylation of p62 and virophagy in vivo require CDKL5.

**Figure 8:**
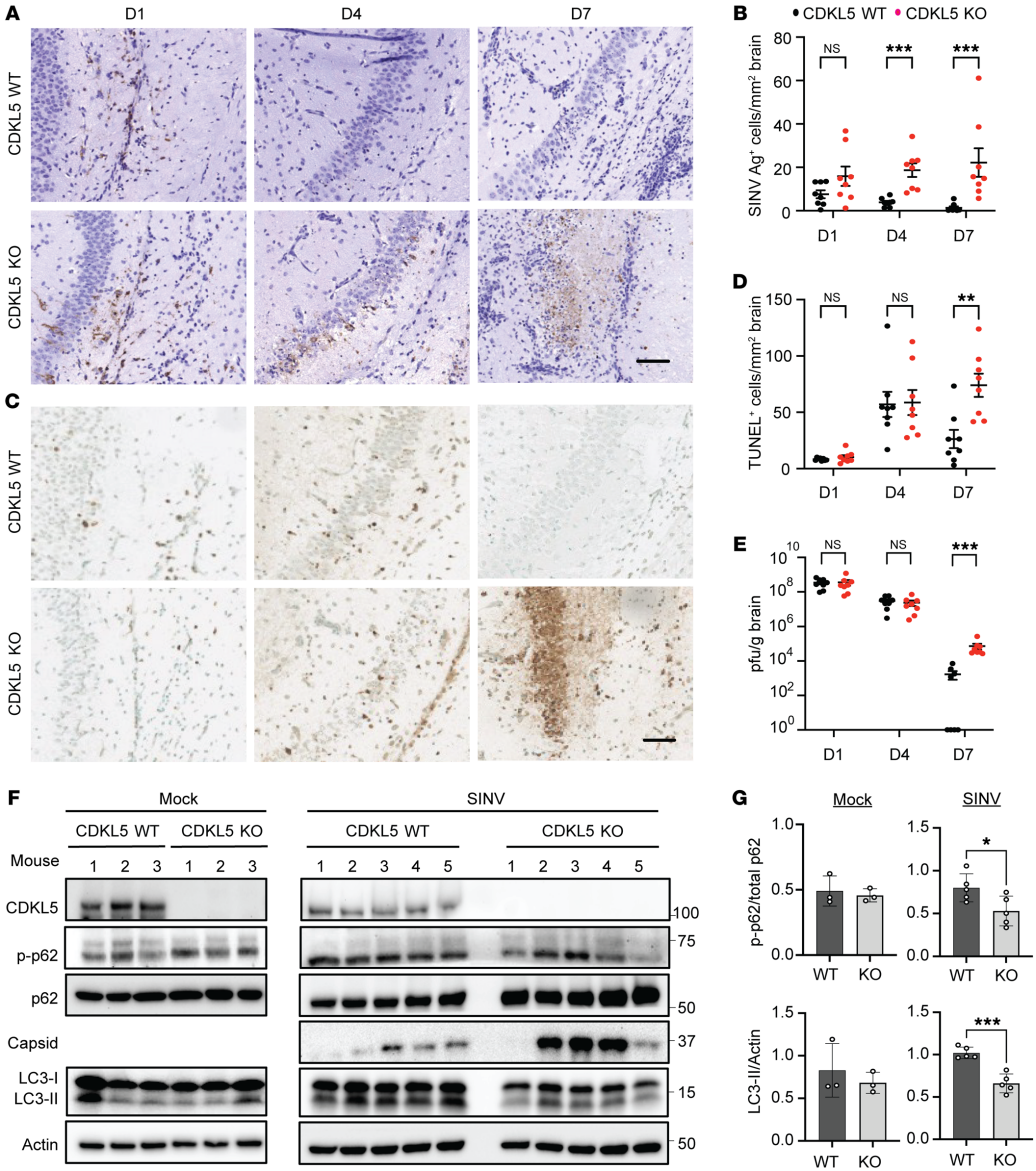
CDKL5 is required for SINV virophagy in vivo. Seven-day-old CDKL5-WT and CDKL5-KO mice were infected intracerebrally with SINV strain dsTE12Q (1 × 10^3^ PFU). (**A**–**E**) Brains were harvested on the indicated days after infection. (**A**) The presence of SINV capsid–positive cells was determined by immunohistochemistry. Representative light micrographs of capsid by immunohistochemistry. Scale bar: 100 μm. (**B**) Quantification of capsid^+^ (Ag^+^) cells per section of brain. Bars represent mean ± SEM of 7–8 mice per group. (**C**) Identification of apoptotic cells in brains sections using TUNEL assay. Representative light micrographs. Scale bar: 200 μm. (**D**) Quantification of TUNEL-positive cells per area of brain. Bars represent mean ± SEM of 7–8 mice per group. (**E**) Viral titers from brain homogenates processed on the indicated days after infection. Bars represent mean ± SEM of 7–8 mice per group. (**F**) Brains from mice either mock inoculated (HBSS) or inoculated with SINV were harvested 7 days after infection. Brain homogenates were subjected to immunoblotting using the indicated antibodies. (**G**) Densitometry analysis of individual mice of p-T269/S272 normalized to total p62 and LC3-II normalized to actin. Dots represent individual mice. Bars on graph represent mean ± SEM of 3 or 5 mice per genotype for control and infected mice, respectively. All *P* values were determined by Mann-Whitney *U* test. *P < 0.05, **P < 0.01, ***P < 0.001.

To gain further insight into the mechanism contributing to the enhanced mortality of SINV-infected CDKL5-KO mice (Figure 1), we explored whether defective capsid virophagy and neuronal cytotoxicity in the absence of CDKL5 occurred in vivo. Following intracranial infection of neonatal WT or CDKL5-KO mice with SINV, we observed that brains of mice lacking CDKL5 had progressively increasing levels of capsid detected by immunohistochemistry (Figure 8, A and B) and neuronal cell death by TUNEL staining (Figure 8, C and D). Conversely, WT mice showed an opposite trajectory, with both clearance of capsid (Figure 8, A and B) and apoptotic cells (Figure 8, C and D) by day 7. Despite these divergent histopathological findings, viral titers in mouse brains on days 1 and 4 were similar between mouse genotypes, indicating that the increased capsid level on day 4 in CDKL5-KO mice was not from an increased early production of infective virions (Figure 8E). By day 7, brains from surviving CDKL5-KO mice demonstrated higher viral titers compared with WT mice (Figure 8E). Tissue cytokine analysis showed that on day 7, interferon-β (IFN-β), critical for host antiviral innate immune response, increased in CDKL5-WT mice, but not in CDKL5-KO mice (Supplemental Figure 9), indicating that an impaired type I IFN response in CDKL5-KO mice later during infection coincides with higher viral replication. In conclusion, these data support the hypothesis that CDKL5 facilitates autophagic clearance of viral antigens, maintenance of cellular and tissue viability, and type I IFN responses after SINV infection.

Because autophagy regulates viral antigen clearance, cellular survival, and type I IFN responses (4), we investigated whether SINV-infected CDKL5-KO mice exhibited defective autophagy at 7 days after infection when differences in response to infection were most apparent between WT and KO mice. We inoculated 7-day-old CDKL5-WT and CDKL5-KO mice with SINV, harvested brains from mock-infected and SINV-infected mice 7 days after infection, and assessed autophagy through Western blot analysis. At baseline, mock-infected CDKL5-WT and CDKL5-KO mice displayed no differences in p-p62, total p62, or LC3-II levels, suggesting similar basal autophagy in the brain (Figure 8, F and G). Conversely, brains from SINV-infected CDKL5-KO mice had a decreased ratio of p-p62 to total p62, decreased LC3-II, increased total p62, and 3 of the 5 mice robustly accumulated capsid (Figure 8, F and G), consistent with defective virophagy and suggestive of an in vivo role for CDKL5 in regulating p62-mediated autophagic clearance of viral antigens.

## Discussion

Here, we report an autophagic function for CDKL5, specifically as a regulator of the selective autophagy of viruses. Our results demonstrate that CDKL5 has a cytoprotective role in SINV-infected cultured cells by facilitating the autophagic clearance of viral proteins. Mechanistically, we identify the selective autophagy receptor p62 as an interacting partner and substrate of CDKL5, and we provide evidence that CDKL5 facilitates the interaction between SINV capsid and p62. In addition to the in vitro findings, we show that CDKL5 is an essential factor in host antiviral defense against diverse viruses by demonstrating that CDKL5-KO mice have enhanced mortality after infection with SINV, CHIKV, and HSV-1.

CDKL5 is a serine/threonine kinase important in neurodevelopment, as loss-of-function mutations in humans result in a severe X-linked neurodevelopmental encephalopathy called CDKL5 deficiency disorder (CDD) (32). By using a combination of genetically ablated cell lines and mouse primary cortical neurons, we demonstrate that CDKL5 is required for the induction of autophagy during SINV infection, but not canonical autophagy induced by nutrient stress. Moreover, a human disease variant of CDKL5, KD CDKL5, failed to rescue autophagy-mediated viral capsid clearance, indicating that CDKL5’s selective autophagy regulatory function depends on its kinase activity. These findings raise the possibility that defective autophagy contributes to the neurological pathogenesis of CDD.

The molecular mechanisms underlying the pathogenesis of CDD and the phosphorylation targets of CDKL5 are not well defined. Notably, several neurological pathologies ranging from neurodegenerative to neurodevelopmental disorders are associated with defective autophagy (2). Failure of selective autophagy to clear protein aggregates or damaged mitochondria has been recognized as a pathologic mechanism of neurodegeneration in Alzheimer disease, Parkinson disease, and amyotrophic lateral sclerosis (33). Likewise, dysfunctional mitochondrial clearance (mitophagy) is implicated in the neurodevelopmental disorder Rett syndrome, which shares similar clinical features to those of CDD (34–36). Thus, selective autophagy, mediated in part by CDKL5, is critical for normal human development, neuronal homeostasis, and the protective response to cellular stressors like organellar damage and infection.

In selective autophagy, the discriminate targeting of cargo to the growing autophagosome depends on autophagy receptors (24). Receptor-cargo recognition followed by recruitment of cargo to autophagosomes involves numerous regulatory proteins, particularly kinases and ubiquitin ligases, which make posttranslational modifications (PTMs) on autophagy receptors and cargo. While the core autophagy receptor p62 is known to interact with SINV capsid (5), the precise molecular trigger that initiates engagement of p62 with capsid was not known. Here, we demonstrate that SINV capsid becomes ubiquitinated, an important signal for p62 recognition of autophagic cargo. Furthermore, we identify that CDKL5 functions as a regulator of p62 cargo recognition by enhancing the interaction between p62 and SINV capsid through phosphorylation of p62 at T269/S272. Notably, p62 serves as a virophagy receptor for capsid proteins from viruses spanning genetic classes that include the double-stranded RNA virus avibirnavirus, the positive-sense single-stranded RNA viruses coxsackievirus, CHIKV, poliovirus, and foot-and-mouth disease virus, and the DNA virus human cytomegalovirus (37–42). The divergent life cycles of these viruses suggest that a shared virus-mediated signal, such as proteotoxic stress from large aggregates of viral antigens, triggers capsid recognition by p62. While the prevalence of viral infections in patients with CDD has not been well studied, an increased incidence of respiratory infections, tonsillitis, ear infections, and urinary tract infections in CDD patients was reported (43), although whether an impaired innate immune response plays a role has yet to be reported. In our animal studies, we demonstrate that CDKL5-KO mice are more susceptible to infection with several genetically diverse viruses compared with their WT littermates. Thus, our findings suggest that CDKL5 may serve as a master regulator of p62-mediated autophagic clearance of viral antigens from multiple classes of viruses through the phosphorylation of p62.

The p62 polypeptide undergoes a variety of PTMs, with phosphorylation being the most common and abundant, to direct its many cellular functions ranging from regulating proinflammatory signaling pathways to delivering cargo to autophagosomes and proteasomes (24). The wide array of p62 cellular functions is attributed to the ability of p62 to assemble into different oligomeric structures ranging from homodimers to large cytoplasmic inclusion bodies (24). Formation of large p62 inclusion bodies results in association with ubiquitinated cargo and trafficking to developing autophagosomes (30, 31, 44). Our studies show that in the absence of CDKL5, phosphorylation of T269/S272 is markedly diminished and p62 less efficiently forms large cytoplasmic inclusion bodies or interacts with SINV capsid. In fact, expression of KD CDKL5 in cells leads to diminished binding between p62 and ubiquitinated substrates during SINV infection, suggesting that CDKL5 kinase activity augments p62’s cargo-binding function during viral infection. Additionally, the expression of a phosphomimetic mutant of p62^T269E/S272D^ in CDKL5-KO cells rescues capsid binding and degradation, suggesting that phosphorylation of these residues plays an important role in the clustering of p62 with capsid cargo followed by autophagic degradation. This result also highlights the critical importance of this phosphorylation event in the context of infection.

How does p62 T269/S272 phosphorylation by CDKL5 affect cell-autonomous innate immunity against viruses? One possible explanation relates to the role of p62 in response to the accumulation of misfolded proteins. Proteotoxic stress due to proteasome inhibition induces p38 MAPK–mediated p62 phosphorylation at T269/S272 to activate the coalescing of p62 microaggregates into large perinuclear bodies to eliminate misfolded proteins through autophagy (28). Large quantities of viral antigens that accumulate during active viral replication also generate proteotoxic stress (45). Therefore, the accumulation of viral protein aggregates may trigger CDKL5-mediated phosphorylation of p62 at T269/S272, activating autophagic clearance of accumulating viral antigens. Remaining critical questions are what specific signals activate CDKL5 to phosphorylate p62 and whether CDKL5 phosphorylates other residues on p62.

Our finding that CDKL5 virophagy function is essential for cellular and organismal survival despite minimal impact on viral replication shifts the paradigm that the specific activity of a host antiviral factor necessitates the inhibition of viral replication. Rather, host antiviral molecules can also function to promote cellular homeostasis in the face of extreme cellular stress. Outside the context of infection, maintenance of neuronal proteostasis requires p62-mediated selective autophagy to prevent the adverse accumulation of protein aggregates and neuronal loss (46, 47). Given that loss-of-function mutations in CDKL5 impact neurodevelopment (32), we speculate that CDKL5 links the cellular antiviral response to accumulating viral antigens with the regulatory network that controls autophagy activation during cellular stress to maintain homeostasis.

In summary, our results reveal that CDKL5 functions as a regulator of virophagy and demonstrate the importance of CDKL5 in host immunity to neurotropic DNA and RNA viruses. Both in vivo and in vitro, CDKL5 prevents the toxic accumulation of viral antigens and promotes cell survival during viral infection. The identification of CDKL5 and its kinase activity as critical for the interaction between SINV capsid and a key selective autophagy receptor, p62, suggests potential targets for therapeutics aimed at increasing antiviral autophagy.

## Methods

Additional details are available in Supplemental Methods.

### Cell culture.

HeLa cells were purchased from American Type Culture Collection (ATCC) and all clones were cultured in reduced-serum Opti-MEM I supplemented with 5% FBS, 100 units/mL penicillin, and 100 μg/mL streptomycin, all purchased from Thermo Fisher Scientific. Cell authentication was performed by the ATCC Cell Line Authentication Service. Two clones of HeLa CRISPR CDKL5-KO cell lines were generated through CRISPR/Cas9–based modification at the Genome Engineering and iPSC Center at Washington University School of Medicine (St. Louis, Missouri, USA). Two guide RNAs were used, 5′-TACCTTCACCTACAACCCCANGG-3′ and 5′-TTTGAGATCCTTGGGGTTGTNGG-3′, to induce a double-strand break in exon 2. KO clones underwent targeted deep sequencing to assess for the presence of insertions and out-of-frame indels. To generate HeLa cells stably expressing GFP-LC3, cells were transfected with pIRES.GFP-LC3.puro plasmid followed by puromycin selection (1 μg/mL). ATG7-KO HeLa cells have been previously described (48) and were a gift from Herbert Virgin (Washington University, St. Louis, Missouri, USA). Vero cells used for plaque assays were cultured in Dulbecco’s modified Eagle medium (DMEM) (Thermo Fisher Scientific) supplemented with 10% FBS, 100 units/mL penicillin, and 100 μg/mL streptomycin.

### Mouse strains.

C57BL/6J (stock 000664) and CDKL5-KO mice (stock 021967) (49) backcrossed more than 10 generations with C57BL/6J were obtained from The Jackson Laboratory. CDKL5-KO mice were additionally backcrossed for 3 generations with C57BL/6J breeders to achieve backcrossing to more than 12 generations. GFP-LC3–transgenic mice have been previously described (50) and were a gift from Noboru Mizushima (University of Tokyo, Tokyo, Japan) and were crossed with CDKL5-KO mice. All mice were housed in a pathogen-free facility with a 12-hour light/dark cycle and fed ad libitum. Age of mice used for specific experiments is indicated in the figure legends.

### Preparation of primary cortical neurons.

To generate mouse cortical neurons, *Cdkl5*^+/–^ female and *Cdkl5*^+/Y^ male or *Cdkl5*^+/–^ female and *Cdkl5*^+/Y^/GFP-LC3^+/+^ male mice were mated and the brains from littermate offspring harvested on embryonic day 15 (E15). The cortex was dissected from the rest of the brain under a dissecting microscope. Cortical cells were dissociated in 2.5% Trypsin (Thermo Fisher Scientific) and DNase I (Sigma-Aldrich), and resuspended in DMEM supplemented with 10% FBS, 100 units/mL penicillin, and 100 μg/mL streptomycin. Cells were then plated on Nunc Lab-Tek II 8-well glass chamber slides (Thermo Fisher Scientific), or 12-well dishes coated overnight with 0.1% polyethyleneimine from Sigma-Aldrich. After 2 hours, the medium was replaced with Neurobasal medium supplemented with B27 (Invitrogen), 2 mM glutamine (Thermo Fisher Scientific), and 5 μM cytarabine (AraC) (Sigma-Aldrich) for 24 hours to suppress growth of non-neuronal cells, at which point fresh medium without AraC was added to culture neurons for 7 days.

### Virus strains.

SINV strain SVIA (ATCC) was derived from a low-passage isolate of the WT SINV strain AR339 (51). Recombinant SINV strain dsTE12Q engineered with a double subgenomic promoter was previously described (6). Recombinant SINV strain dsTE12Q.mCherry-capsid (SINV.mCherry-capsid) was previously described (5). The mutant HSV-1 strain (HSV-1ΔBBD) was previously described (14). The CHIKV strain 06-021 was a gift from Deborah J. Lenschow (Washington University, St. Louis, Missouri, USA). Viral stocks were propagated and titrated by plaque assays in either Vero or BHK-21 cells. The SINV strain SVIA was used in all infection studies with HeLa cells and primary cortical neurons unless otherwise specified in the figure legends.

### Statistics.

All statistical analyses were performed using GraphPad Prism software (version 9). The individual statistical tests used are indicated on the figure legends. For in vitro studies, data were analyzed using unpaired, 2-tailed *t* test when comparing 2 groups and analysis of variance (ANOVA) when comparing more than 2 groups, all under the assumption of normality. Mann-Whitney *U* test was used for a nonparametric comparison between 2 groups of mice when analyzing in vivo pathogenesis data. For mouse mortality studies, Kaplan-Meyer survival curves were generated and analyzed through log-rank tests to determine statistical significance.

### Study approval.

Mouse experiments were reviewed and approved by the Institutional Animal Care and Use Committee at the University of Texas Southwestern and followed the NIH *Guide for the Care and Use of Laboratory Animals* (National Academies Press, 2011). The University of Texas Southwestern is accredited by the American Association for Accreditation of Laboratory Animal Care (AAALAC).

### Data availability.

The macro used for puncta counting and image analysis through FIJI3 software can be downloaded from GitHub (https://github.com/thinwaj/Puncta-quantification-with-Fiji). All data used to generate graphs are available in the Supporting Data Values file. Request for reagents and protocols should be directed to the corresponding author, JWT.

## Author contributions

JWT, BL, JLJ, JKP, TAR, and MUS conceptualized the study. JWT, TAR, and MUS analyzed the data. JWT, BL, and TAR acquired funding. JWT, ZZ, EP, SS, KH, YW, VS, MG, and GU carried out the investigation. JWT, MUS, and TAR provided project administration. JWT, BL, JLJ, JKP, TAR, and MUS supervised the project. JWT, TAR, and MUS generated figures. JWT, JKP, TAR, and MUS wrote the original draft of the manuscript, which was reviewed and edited by all authors.

## Supplementary Material

Supplemental data

Supporting data values

## Version 1 11/02/2023

In-Press Preview

## Version 2 01/02/2024

Electronic publication

## References

[1] Ahmad L (2018). Autophagy-virus interplay: from cell biology to human disease. Front Cell Dev Biol.

[2] Levine B, Kroemer G (2019). Biological functions of autophagy genes: a disease perspective. Cell.

[3] Mizushima N, Levine B (2020). Autophagy in human diseases. N Engl J Med.

[4] Viret C (2021). Selective autophagy receptors in antiviral defense. Trends Microbiol.

[5] Orvedahl A (2010). Autophagy protects against Sindbis virus infection of the central nervous system. Cell Host Microbe.

[6] Liang XH (1998). Protection against fatal Sindbis virus encephalitis by Beclin, a novel Bcl-2-interacting protein. J Virol.

[7] Dong X (2021). Sorting nexin 5 mediates virus-induced autophagy and immunity. Nature.

[8] Bahi-Buisson N, Bienvenu T (2012). CDKL5-related disorders: from clinical description to molecular genetics. Mol Syndromol.

[9] Rusconi L (2008). CDKL5 expression is modulated during neuronal development and its subcellular distribution is tightly regulated by the C-terminal tail. J Biol Chem.

[10] Zhu YC, Xiong ZQ (2019). Molecular and synaptic bases of CDKL5 disorder. Dev Neurobiol.

[11] Klionsky DJ (2021). Guidelines for the use and interpretation of assays for monitoring autophagy (4th edition)^1^. Autophagy.

[12] Bahi-Buisson N (2008). Key clinical features to identify girls with CDKL5 mutations. Brain.

[13] Bertani I (2006). Functional consequences of mutations in CDKL5, an X-linked gene involved in infantile spasms and mental retardation. J Biol Chem.

[14] Orvedahl A (2007). HSV-1 ICP34.5 confers neurovirulence by targeting the Beclin 1 autophagy protein. Cell Host Microbe.

[15] Orvedahl A (2011). Image-based genome-wide siRNA screen identifies selective autophagy factors. Nature.

[16] (2016). Fanconi anemia proteins function in mitophagy and immunity. Cell.

[17] Shoji-Kawata S (2013). Identification of a candidate therapeutic autophagy-inducing peptide. Nature.

[18] Hector RD (2016). Characterisation of CDKL5 transcript isoforms in human and mouse. PLoS One.

[19] Wengler G (1987). The mode of assembly of alphavirus cores implies a mechanism for the disassembly of the cores in the early stages of infection. Brief review. Arch Virol.

[20] Dowdle WE (2014). Selective VPS34 inhibitor blocks autophagy and uncovers a role for NCOA4 in ferritin degradation and iron homeostasis in vivo. Nat Cell Biol.

[21] Kumar AV (2022). Selective autophagy receptor p62/SQSTM1, a pivotal player in stress and aging. Front Cell Dev Biol.

[22] Van Bergen NJ (2022). CDKL5 deficiency disorder: molecular insights and mechanisms of pathogenicity to fast-track therapeutic development. Biochem Soc Trans.

[23] Muñoz IM (2018). Phosphoproteomic screening identifies physiological substrates of the CDKL5 kinase. EMBO J.

[24] Gubas A, Dikic I (2022). A guide to the regulation of selective autophagy receptors. FEBS J.

[25] Matsumoto G (2011). Serine 403 phosphorylation of p62/SQSTM1 regulates selective autophagic clearance of ubiquitinated proteins. Mol Cell.

[26] Pilli M (2012). TBK-1 promotes autophagy-mediated antimicrobial defense by controlling autophagosome maturation. Immunity.

[27] Matsumoto G (2015). TBK1 controls autophagosomal engulfment of polyubiquitinated mitochondria through p62/SQSTM1 phosphorylation. Hum Mol Genet.

[28] Zhang C (2018). p38Δ MAPK regulates aggresome biogenesis by phosphorylating SQSTM1 in response to proteasomal stress. J Cell Sci.

[29] Sarraf SA (2020). Loss of TAX1BP1-directed autophagy results in protein aggregate accumulation in the brain. Mol Cell.

[30] Zaffagnini G (2018). p62 filaments capture and present ubiquitinated cargos for autophagy. EMBO J.

[31] Wurzer B (2015). Oligomerization of p62 allows for selection of ubiquitinated cargo and isolation membrane during selective autophagy. Elife.

[32] Katayama S (2020). Cyclin-dependent kinase-like 5 (CDKL5): possible cellular signalling targets and involvement in CDKL5 deficiency disorder. Neural Plast.

[33] Fleming A (2022). The different autophagy degradation pathways and neurodegeneration. Neuron.

[34] Crivellari I (2021). Impaired mitochondrial quality control in Rett syndrome. Arch Biochem Biophys.

[35] Sbardella D (2017). Retention of mitochondria in mature human red blood cells as the result of autophagy impairment in Rett syndrome. Sci Rep.

[36] Kadam SD (2019). Rett syndrome and CDKL5 deficiency disorder: from bench to clinic. Int J Mol Sci.

[37] Li Y (2020). Cytoplasmic cargo receptor p62 inhibits avibirnavirus replication by mediating autophagic degradation of viral protein VP2. J Virol.

[38] Mohamud Y (2019). CALCOCO2/NDP52 and SQSTM1/p62 differentially regulate coxsackievirus B3 propagation. Cell Death Differ.

[39] Judith D (2013). Species-specific impact of the autophagy machinery on chikungunya virus infection. EMBO Rep.

[40] Berryman S (2012). Foot-and-mouth disease virus induces autophagosomes during cell entry via a class III phosphatidylinositol 3-kinase-independent pathway. J Virol.

[41] Zimina A (2021). Interaction of poliovirus capsid proteins with the cellular autophagy pathway. Viruses.

[42] Zimmermann C (2021). Autophagy interferes with human cytomegalovirus genome replication, morphogenesis, and progeny release. Autophagy.

[43] Mangatt M (2016). Prevalence and onset of comorbidities in the CDKL5 disorder differ from Rett syndrome. Orphanet J Rare Dis.

[44] Bjorkoy G (2005). p62/SQSTM1 forms protein aggregates degraded by autophagy and has a protective effect on huntingtin-induced cell death. J Cell Biol.

[45] Zhang L, Wang A (2012). Virus-induced ER stress and the unfolded protein response. Front Plant Sci.

[46] Hetz C (2021). Adapting the proteostasis capacity to sustain brain healthspan. Cell.

[47] Menzies FM (2015). Compromised autophagy and neurodegenerative diseases. Nat Rev Neurosci.

[48] Selleck EM (2015). A noncanonical autophagy pathway restricts toxoplasma gondii growth in a strain-specific manner in IFN-γ-activated human cells. mBio.

[49] Wang IT (2012). Loss of CDKL5 disrupts kinome profile and event-related potentials leading to autistic-like phenotypes in mice. Proc Natl Acad Sci U S A.

[50] Mizushima N (2004). In vivo analysis of autophagy in response to nutrient starvation using transgenic mice expressing a fluorescent autophagosome marker. Mol Biol Cell.

[51] Taylor RM (1955). Sindbis virus: a newly recognized arthropodtransmitted virus. Am J Trop Med Hyg.

