# Supplementary material for: CDKL5 regulates p62-mediated selective autophagy and confers protection against neurotropic viruses
Source: J Clin Invest. 2024 Jan 2;134(1):e168544. doi: 10.1172/JCI168544 (PMC10760973; doi:10.1172/JCI168544)
Supplement: Supplemental data [file jci-134-168544-s197.pdf]

Supplemental Information

CDKL5 regulates p62-mediated selective autophagy and confers protection against neurotropic viruses

Josephine W. Thinwa, Zhongju Zou, Emily Parks, Salwa Sebti, Kelvin Hui, Yongjie Wei, Mohammad Goodarzi, Vibha Singh, Greg Urquhart, Jenna L. Jewell, Julie K. Pfeiffer, Beth Levine, Tiffany A. Reese, and Michael U. Shiloh

Supplementary Methods

Reconstitution of HeLa CDKL5 KO cells with CDKL5 and p62 alleles

Wild type CDKL5 (NM_001323289.2), WT CDKL5-3xFlag, KD CDKL5^{k42R}, KD CDKL5^{K42R}-3xFLAG, WT p62-3xFLAG, p62$^{T269A/S272A}$ -3xFLAG, p62 $^{T269E/S272D}$-3xFLAG, and p62$^{LIR\ mut}$-3xFLAG and p62$^{\Delta UBA}$ -3xFLAG mutants previously described (31) were generated as geneblocks by Integrated DNA Technologies and cloned into pLenti-c-Myc-DDK-IRES-Neo vector (Origene) using NEBuilder® HiFi DNA Assembly Master Mix per manufacturer's instructions (New England BioLabs, E2621S). To generate the lentiviruses, the vectors were transfected into Phoenix cells (ATCC) together with packaging vectors pCMVR8.91 (1) and pMDG (2). Cell culture supernatants were collected on days 2 and 3, filtered with a 0.45 μm filter (EMD Millipore) and then used to infect WT or CDKL5 KO HeLa cells in the presence of 8 μg/ml polybrene (SCBT, sc-134220). After 6 h, virus-containing media was exchanged with fresh culture media. After 48 h, 500 μg/ml of geneticin (500ng/ml) (Thermo Fisher, 10131027) was added for a 7-day selection period and continued at 100 μg/ml for an additional 14 days prior to SINV experiments.

Assays for autophagy assessment

To determine the induction of autophagy in HeLa cells and primary cortical neurons (3), the numbers of autophagosomes as represented by GFP-LC3 puncta per cells were counted by an observer blinded to the condition or cell genotype by fluorescence microscopy. In addition, LC3-I to LC3-II conversion was determined by western blot using anti-LC3B antibody. For virophagy experiments, cells were infected with SINV strains MOI of 10 in Opti-MEM I Reduced Serum Medium supplemented with 5% FBS (culture medium) for 1h then exchanged with fresh culture media or maintained in culture media only (mock). For amino-acid starvation, cells were treated

for either 1h (GFP-LC3 microscopy assays) or 3 h (LC3 I/II western blots) with Earle's Balanced Salt Solution (EBSS, Thermo Fisher Scientific) at 37°C and for mTOR inhibition, with 250 nM of torin 1 (Selleck Chemicals S2827) or DMSO as the vehicle control for the same periods of time. Autophagic flux was assessed by exposing cells to BafA1 (100nM) for 2h.

Antibodies

The following antibodies were used: rabbit anti-LC3B (Novus Biologicals, NB100-2220), guinea pig anti-p62/SQSTM1 (C-terminus) (Progen, GP62-C), mouse anti-SQSTM1 (Abnova, H00008878-M01), rabbit anti-phospho-SQSTM1/p62 (Thr269/Ser272) (Cell Signaling Technology, 13121), rabbit anti-phospho-SQSTM1/p62 (Ser403) (Cell Signaling Technology, 39786), mouse anti-actin antibody (C4) HRP (Santa Cruz Biotechnology (SCBT), sc-47778 HRP), mouse anti-CDKL5 (D-12) antibody (SCBT, sc-376314), rabbit anti-ATG7 polyclonal antibody (Sigma-Aldrich, A2856), rabbit anti-TBK1/NAK antibody (Cell Signaling Technology, 3013), rabbit anti SINV capsid antibody (Gift from Diane Griffin) (4), rabbit anti-HA tag (C29F4) antibody (Cell Signaling Technology, 3724), rat anti-HA (Sigma-Aldrich, 11867423001), mouse anti-NBRI antibody, clone 6B11 (Abnova, H00004077-M01), rabbit anti-NDP52 antibody (Cell Signaling Technology, 60732S), rabbit anti-OPTN antibody (Proteintech, 10837-1-AP), rabbit anti-TAX1BP1 antibody (4H2L18) (Thermo Fisher, 702840), mouse anti-Flag M2 antibody (Sigma, F1804), rabbit anti-Lamp1 (Cell Signaling Technology, 9091), mouse anti alpha-tubulin (Sigma-Aldrich, T5168), rabbit anti-Histone H3 (Cell Signaling Technology, 9715S), rabbit anti-ubiquitin (Cell Signaling Technology, 43124), donkey anti-rabbit IgG HRP-conjugate species-adsorbed (Millipore Sigma, AP182P), goat anti-mouse IgG (H+L) HRP (Millipore Sigma, AP308P), donkey anti-mouse IgG (H+L) highly cross-adsorbed secondary antibody Alexa Fluor 488 (Thermo Fisher Scientific, A21202), donkey anti-rabbit IgG (H+L) highly cross-adsorbed

secondary antibody Alexa Fluor 488 (Thermo Fisher Scientific, A-21206), and donkey anti-rabbit IgG (H+L) highly cross-adsorbed secondary antibody Alexa Fluor 594 (Thermo Fisher Scientific, A21207).

Generation of dsTE12Q.HA-capsid virus

For the generation of dsTE12Q.HA-capsid virus, a geneblock fragment "CATCTGACTAATACTACAACACCACCACCATGTACCCGTATGATGTTCCGGATTAC GCTGGCTATCCCTACGACGTGCCCGACTATGCCGGGTACCCCTATGACGTCCCAGAC TACGCA AATAGAGGATTCTTTAACATGCTCGGC" containing a partial capsid sequence and the in-frame 3xHA coding sequence was synthesized (IDT). The dsTE12Q recombinant vector was linearized by PCR with primers "AATAGAGGATTCTTTAACATGCTCGGC" and "GGTGGTGGTGTTGTAGTATTAGTCAGATG" immediately after the capsid start codon. The linearized vector and geneblock were then mixed to create the dsTE12Q.HA-Capsid recombinant SINV vector using NEBuilder® HiFi DNA Assembly Master Mix per manufacturer's instructions (New England BioLabs, E2621S).

Infection with UV-inactivated SINV

To generate UV-inactivated virus, SINV strain SVIA equivalent to MOI of 500 was irradiated for 7 minutes using a Stratalinker UV Crosslinker 1800 (Stratagene). Absence of infectious particles was confirmed through plaque assay on Vero cells. After UV-inactivation, HeLa cells were exposed to virus particles for 1 h and then washed three times to remove extracellular or loosely bound virus before fresh culture media was added. Lysates were harvested for western blot analysis at timed intervals to chase the clearance of capsid protein. To block autophagy induction and flux, 5 μM of PIK-III inhibitor (Selleck Chemicals, S7683), 100nM of

Bafilomycin A1 (Sigma-Aldrich B1793), or 50nM of Epoxomicin (Selleck Chemicals, S7038) were added 30 minutes after cells were exposed to UV-inactivated SINV.

Fluorescence microscopy

For all cell culture microscopy experiments, HeLa cells and cortical neurons were cultured on Nunc Lab-Tek II 4 or 8-well glass chamber slides (Thermo Fisher Scientific). After exposure to autophagy inducing conditions, cells were fixed for 7 minutes with 4% PFA in PBS at room temperature and washed three times with PBS. For detection of GFP-LC3 or mCherry-capsid, a coverslip was mounted onto each slide with VECTASHIELD Antifade Mounting Medium with DAPI (Vector Laboratories H-1200), the perimeter of the coverslip was sealed with nail polish and allowed to dry overnight at room temperature before imaging. For immunofluorescence, both HeLa cells and cortical neurons were permeabilized after PFA fixation with 100% methanol chilled at -20°C for 20 minutes followed by 1 h blocking with PBS containing 3% BSA (Sigma-Aldrich; A9418). Cells were stained with antibodies against SIN capsid, SQSTMI/p62 and phospho-SQSTM1/p62 for 2 h at room temperature in blocking buffer. After three washes, cells were treated with Alexa Fluor secondary antibodies for 1 h at room temperature, washed three times, and then mounted. All fluorescence imaging was performed using a Zeiss AxioImager Z2 microscope equipped with a Photometrics CoolSnap HQ2 CCD camera using a Zeiss PLAN APOCHROMAT 20X/0.8 NA wide-field objective or PLAN APOCHROMAT 40X/0.9 NA oil immersion objective and a Nikon CSU-W1 SoRa spinning disk confocal microscope. In all experiments a secondary antibody control was used for thresholding the background. Images were taken randomly of areas with similar cell density with the experimenter blinded to the experimental condition. HeLa cells SINV capsid/p62 and phospho-p62/total p62 images were taken in 0.3um Z-stacks and deconvoluted with AutoQuant X3 software. Image analysis of the p62 inclusion bodies

was performed using Fiji software through a custom-written macro. LAMP1 and capsid colocalization analysis was performed through Imaris software.

Transmission electron microscopy

CDKL5 WT and KO HeLa cells were infected with HSV-1 ΔBBD strain at an MOI of 10 for 12 hours. Cells were fixed for 10 minutes with 2.5% glutaraldehyde + 2% Paraformaldehyde in 0.1M cacodylate buffer. Fixed samples were washed in 0.1 M sodium cacodylate buffer and treated with 0.1% Millipore-filtered cacodylate buffered tannic acid, postfixed with 1% buffered osmium tetroxide, and stained en bloc with 1% Millipore-filtered uranyl acetate. The samples were dehydrated in increasing concentrations of ethanol, infiltrated, and embedded in LX-112 medium. The samples were polymerized in a 60°C oven for approximately 3 days. Ultrathin sections were cut in a Leica Ultracut microtome (Leica, Deerfield, IL), stained with uranyl acetate and lead citrate and examined in a JEM 1010 transmission electron microscope (JEOL, USA, Inc., Peabody, MA) at an accelerating voltage of 80 kV. Digital images were obtained using AMT Imaging System (Advanced Microscopy Techniques Corp, Danvers, MA).

SINV growth curves

To perform high MOI or low MOI multi-step viral growth curve analysis, HeLa cells were cultured in triplicate on 6-well plates and infected with either MOI of 10 or 0.01, respectively for 1.5 h. Virus infection media was replaced with fresh culture media. One hundred microliter samples were drawn from each well at specified time intervals and virus titers determined through Vero cell plaque assays.

Cell death assay

For measurement of virus-induced cell death, cells were mock-infected or infected with SINV (MOI 10) for 24 h and then processed using the CellTiter-Glo Luminescent Cell Viability Assay, per manufacturer's instructions (Promega, G7570). Luminescence was detected using a CLARIOstar Plate Reader (BMG Labtech).

Co-immunoprecipitation

To investigate the interactions between CDKL5, p62 and SINV capsid, 10^7 HeLa cells were cultured on 150 mm plates and either mock-infected or infected for 7 h at MOI of 10 with recombinant SINV expressing HA-capsid. HeLa cells were scrapped from the plate and lysed in ice-cold lysis buffer (50 mM Tris-HCL (pH 7.5), 150 mM NaCl, 1 mM EDTA, 1% Triton X-100) containing complete proteinase inhibitor cocktail (Roche) and Halt phosphatase inhibitor cocktail (Thermo Fisher Scientific) for 30 min on ice. Cellular debris was removed with centrifugation for 10 minutes at 15,000 g at 4°C. Supernatants were pre-cleared with either dynabeads protein G (Thermo Fisher; 10003D) or protein G PLUS agarose beads (SCBT, sc-2002) for 1.5h at 4°C with gentle agitation. After pelleting the beads, supernatants were mixed with either 0.8 μg/mL of rat anti HA antibody (Sigma-Aldrich, 11867423001) and 30 μL of pre-washed dynabeads protein G (Thermo Fisher; 10003D) or 40ul of pre-washed anti-Flag M2 affinity gel (Millipore Sigma; A2220) and then incubated at 4°C overnight with gentle agitation. Dynabeads were pelleted using the DynaMag-2 Magnet (Thermo Fisher, 12321D) and anti-Flag beads by centrifugation at 3000g for 1 minute followed by 3 washes with ice-cold lysis buffer and two additional washes with lysis buffer containing 300mM NaCl. Immunoprecipitated proteins were eluted by adding 2X Laemmli sample buffer (Bio-Rad Laboratories) containing 5% β-mercaptoethanol (Bio-Rad Laboratories) and boiling sample for 7 minutes followed by western blot analysis.

Affinity purification of ubiquitinated capsid using TUBEs

To determine whether SINV capsid is ubiquitinated during HeLa cells infection, we used magnetic bead fused TUBEs (LifeSensors Inc; UM401M) according to manufactures instructions.

Briefly, cell lysates were mixed with 50µL of TUBE or control magnetic beads (LifeSensors Inc; UM500M) and incubated at 4°C for 2 hours. The TUBE and control beads were then washed five times with TBST, resuspended in 2x Laemmli sample buffer, boiled and subjected to SDS-PAGE.

Protein lysate preparation and Western blot analyses

HeLa cells and cortical neurons were lysed with ice-cold 1X RIPA buffer (Cell Signaling Technology, 9806) supplemented with proteinase inhibitor cocktail (Sigma) and Halt phosphatase inhibitor cocktail (Thermo Fisher Scientific) and incubated on ice for 30 min. The insoluble components of the lysates were pelleted by centrifugation at 15,000 g at 4°C for 15 min, and the residual supernatants mixed with 2X Laemmli sample buffer containing 5% β-mercaptoethanol. For analysis of p62 in total lysates, cells were directly lysed in 2x Laemmli sample buffer, boiled for 3 minutes in a 100°C heating block, and then sonicated in an ultrasonic bath for one minute. For protein analysis in the soluble versus insoluble pellet, the pellet was washed with 1X RIPA buffer and resuspended with 2X Laemmli sample buffer at a ratio of 3:1. Nuclear/cytoplasmic fractionations were performed as previously described (5). Proteins were separated on gradient 4-20% Mini-PROTEAN TGX precast protein gels (Bio-Rad Laboratories) and transferred onto PVDF membranes (Bio-Rad Laboratories). For blocking, membranes were incubated in 5% non-fat milk in either 1X TBS or PBS containing 0.01% Tween 20. Primary and secondary antibodies were diluted in blocking buffer. Proteins were detected with SuperSignal West Pico PLUS Chemiluminescent Substrate (Thermo Fisher Scientific) using a Bio-Rad Chemidoc imager. Band densitometry was determined through Fiji software.

Flow Cytometry

WT and CDKL5 KO HeLa cells infected with SINV/mCherry-capsid were dissociated with 0.25% trypsin, fixed with 4% PFA for 5 minutes and resuspended in PBS with 3% BSA. Cells

positive for mCherry-Capsid were detected through an LSRII-HTS flow cytometer (BD Biosciences) and data analyzed using FlowJo software (Version 9).

Quantitative real-time PCR (qRT-PCR)

To assess p62 expression, RNA from HeLa cells infected with SINV was extracted at indicated time points using RNeasy Plus Mini Kit (Qiagen) and reverse-transcribed using the iScript cDNA Synthesis Kit (Bio-Rad Laboratories; 1708891). QuantiFast SYBR Green RT-PCR Kit (Qiagen, 204156) was used for the qPCR reaction and measurement performed on the 7500 Fast Real-Time PCR System (Applied Biosystems). The following primers were used for the reaction:

p62 forward primer: 5'-TACGACTTGTGTAGCGTCTG-3'

p62 reverse primer: 5'-CGTGTTTCACCTTCCGGAG-3'

GAPDH forward primer: 5'- CGTGTCAGTGGTGGACCTG-3'

GAPDH reverse primer: 5'-CGTCAAAGGTGGAGGAGTGG-3'

In vitro Kinase Assays

For the reaction, 0.3 μg of recombinant human CDKL5 (1-498) (ThermoFisher, A33353) was combined with 3 μg of recombinant human SQSTM1/p62 protein (GeneTex, GTX68012-pro) in buffer containing 20 mM HEPES (pH 7.5) and 10 mM $MgCl_2$ in the presence or absence of 50 μM [γ-^{32}P] ATP. The reaction was incubated at 30°C for up to 45 minutes, stopped by adding 4x Laemmli sample buffer, then subjected to SDS-PAGE for Coomassie staining and radioactivity detection on x-ray film. This same reaction was also done using recombinant CDKL5 or TBK-1 (positive control) combined with recombinant p62 protein, with or without 100 μM non-radioactive ATP, and phosphorylation of p62 detected by western blot using phosphospecific antibodies. To compare the ability of WT and KD CDKL5 to phosphorylate recombinant p62

protein at Thr269/Ser272, HeLa cells stably expressing EV, WT or KD CDKL5 were lysed in ice-cold buffer (50 mM HEPES (pH 7.5), 300 mM NaCl, 1% Triton X-100) supplemented with proteinase inhibitor cocktail (Sigma) and Halt phosphatase inhibitor cocktail (Thermo Fisher Scientific). Pull down of FLAG was performed as described above with anti-Flag M2 affinity gel. The beads were washed five times with lysis buffer containing 0.5 M NaCl and then three times with the in vitro kinase buffer (20 mM HEPES (pH 7.5), 10 mM $MgCl_2$, and 1 mM DTT) before resuspending the beads in 20 μl of in vitro kinase buffer with 100 μM ATP. The reaction was incubated at 30°C for 45 minutes. Phosphorylation analysis was performed using Western blot.

Virus infection of mice

Mice were inoculated intracerebrally into the right cerebral hemisphere with SINV and HSV-1 and subcutaneously with CHIKV. Viruses were diluted in Hank's Balanced Salt Solution (HBSS, Thermo Fisher Scientific) and 30μL inoculum was used per mouse. For SINV, the dsTE12Q strain was used to infect seven-day-old *CDKL5*^{WT} and *CDKL5*^{KO} neonates with $1x10^3$ pfu. Separately, seven-day-old neonates were infected with $1x10^5$ pfu of CHIKV strain 06-02. For HSV-1 infection, $5x10^4$ pfu of HSV-1ΔBBD strain was used to infect anesthetized eight- to ten-week-old littermate *CDKL5*^{WT} and *CDKL5*^{KO} mice. For mortality studies, mice were monitored daily for 21 days. To perform brain tissue analysis in SINV infected mice, mice from each genotype were randomly selected for dissection at days 1, 4 and 7. The right hemisphere was snap frozen in liquid nitrogen, homogenized in HBSS and used for plaque assay titration to determine the viral load. Brain homogenates from SINV infected mice were also assessed for IFN-β levels through mouse IFN-β ELISAs per manufacturer's instructions (Abcam, ab252363). The left hemisphere was fixed in 4% PFA, cryoprotected in 30% sucrose and embedded in paraffin for histology studies.

Histology

For in vivo cell death analysis, terminal deoxynucleotidyl transferase dUTP nick end labeling (TUNEL) staining of paraffin-embedded sagittal sections was performed using Apoptag Peroxidase In Situ Apoptosis Detection Kit (EMD Millipore, S7100), per the manufacturers' instructions. For immunohistochemistry, brain sections were stained with rabbit anti-SINV capsid antibody (1:5000 dilution) and the Vectastain Elite ABC HRP kit (Vector Laboratories, PK-6101) was used for signal detection, per manufacturer's instructions. To capture the entire brain section, a Zeiss Axio Scan.Z1 slide scanner equipped with a Zeiss PLAN APOCHROMAT 20X/0.8 NA objective (Carl Zeiss Microscopy, UT Southwestern Whole Brain core facility) was used image each brain section. The number of TUNEL-positive or capsid positive cells per mouse brain sections was counted by an observer blinded to the condition and genotype.

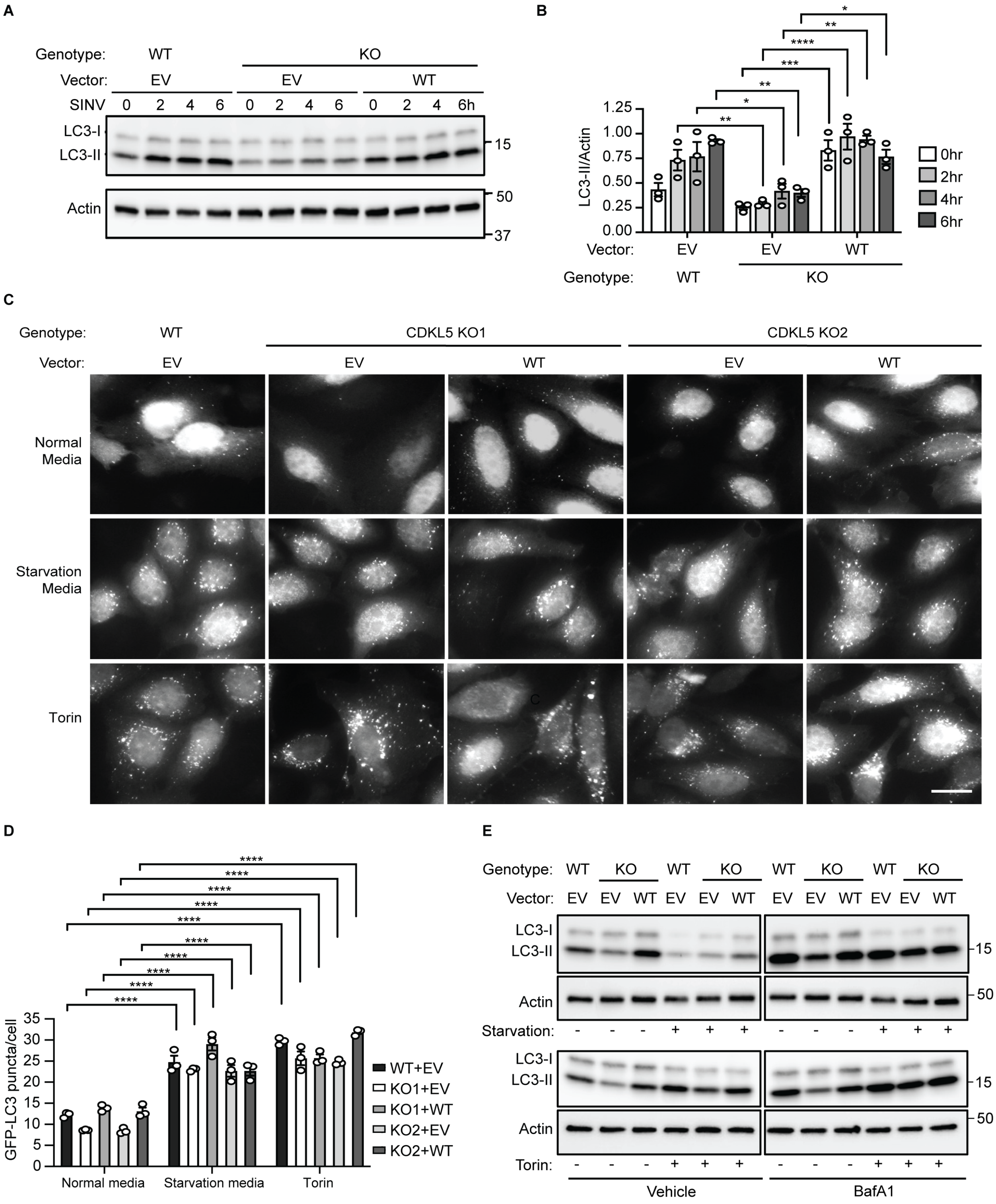

Supplemental Figure 1. CDKL5 regulates basal and virus-induced autophagy, but not torin or amino-acid starvation induced autophagy

(A) Western blot of LC3 conversion over a 6h time course comparing WT and CDKL5 KO HeLa cells reconstituted with WT CDKL5 or EV and infected with SINV (MOI=10).

(B) Quantification of LC3-II signal normalized to actin displayed as mean ± SEM from three independent experiments; significance was assessed using one-way ANOVA with Sidak's multiple comparison test (*$p < 0.05$, **$p < 0.01$, ***$p < 0.001$, and ****$p < 0.0001$).
(C,D) WT and CDKL5 KO/GFP-LC3 HeLa clones reconstituted with EV or WT CDKL5 were treated with normal growth media, EBSS (starvation) medium or Torin (250 nM) for 1h. (C) Representative fluorescent micrographs of GFP-LC3 puncta. Scale bars: 20 μm.
(D) Quantification of puncta per cell. Bars represent mean ± SEM of triplicate samples with at least 100 cells analyzed per sample. Shown is a representative of three independent experiments. This experiment was performed contemporaneously with Figure 1A. Micrographs of normal media samples shown in the top row of Supplemental Figure 1C are identical to those in Figure 1A. GFP-LC3 puncta quantification for normal media shown in Supplemental Figure 1D is identical to normal media quantification in Figure 1C. Statistical analysis, inclusive of the data in Figure 1C, was performed using one-way ANOVA with Sidak's multiple comparison test (****$p < 0.0001$).
(E) Western blot of LC3-I and LC3-II from WT and CDKL5 KO HeLa cells reconstituted with WT CDKL5 or EV and treated with normal media, EBSS medium or Torin for 3h in the presence or absence of BafA1 (100uM) for the final 2 hours. Blot representative of 3 independent experiments.

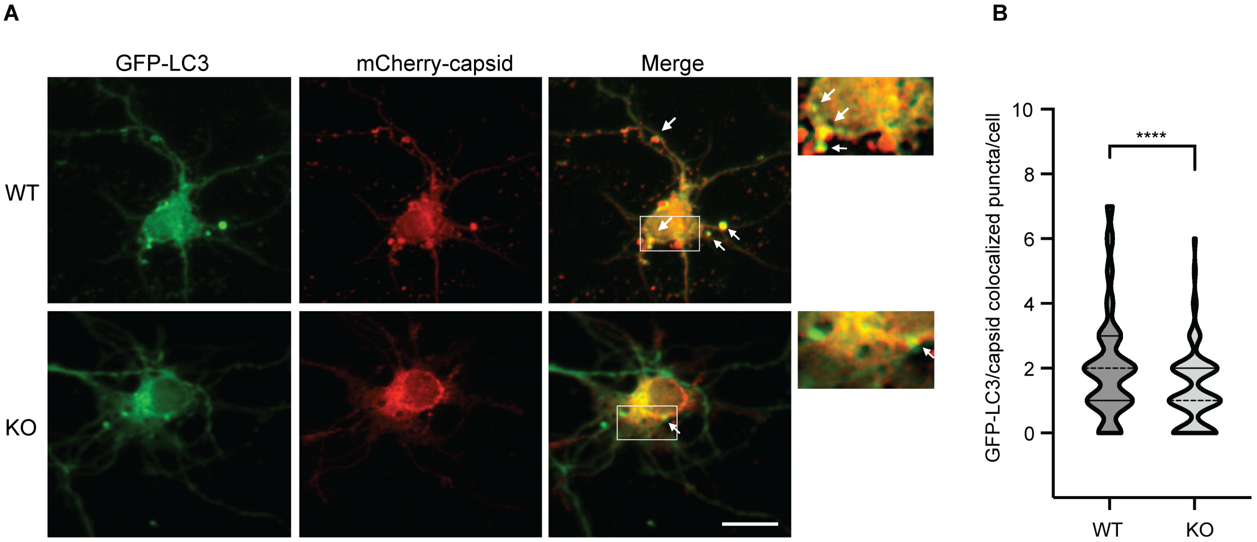

Supplemental Figure 2. SINV-induced autophagy in mouse cortical neurons requires CDKL5

(A) Representative fluorescent micrographs of mCherry capsid and GFP/LC3 colocalization in primary cortical neurons isolated from littermate CDKL5 WT/GFP-LC3 and CDKL5 KO/GFP-LC3 embryos and infected with SINV/mCherry-capsid for 8hours. Arrows indicate representative colocalized puncta. Scale bar: 20 μm.

(B) Quantification of colocalized puncta per cell. Violine plot with dashed line as median and solid lines as first and third quartiles. Over 150 neurons analyzed per condition. *p* values were determined by Welch's unpaired two-tailed t-test (*****p* < 0.0001).

.

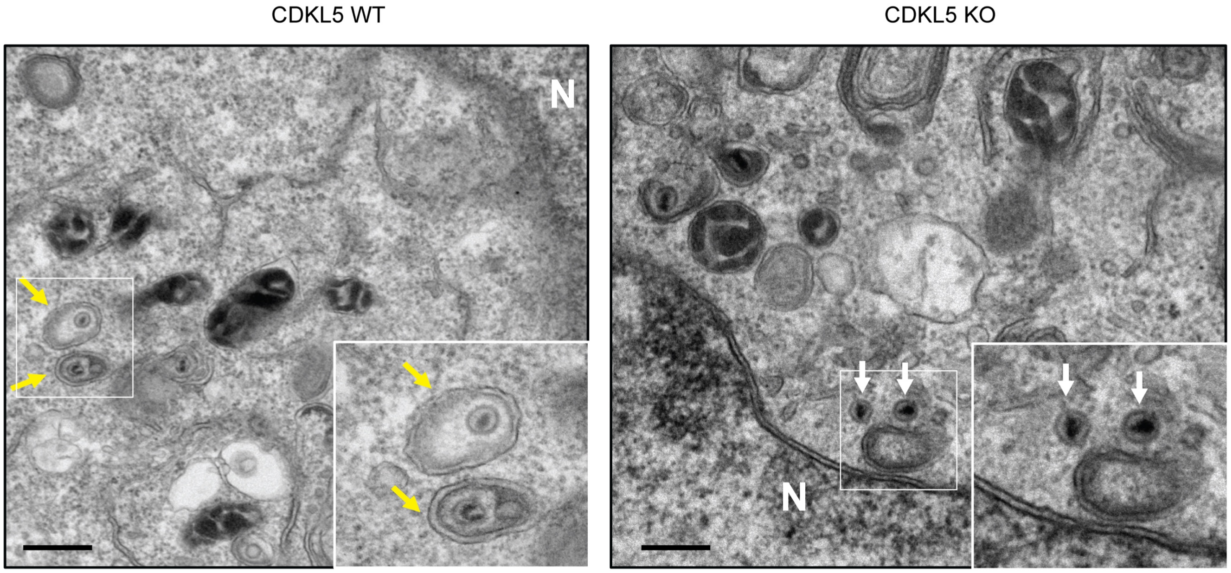

Supplemental Figure 3. CDKL5 functions in HSV-1 virophagy
Electron microscopy images of WT and CDKL5 KO HeLa cells infected with HSV-1 ΔBBD (MOI 10) for 12 hours. Yellow arrows, representative autophagic vacuoles (AVs) containing HSV-1 and white arrows, HSV-1 virions outside AVs. Scale bar: 600nm.

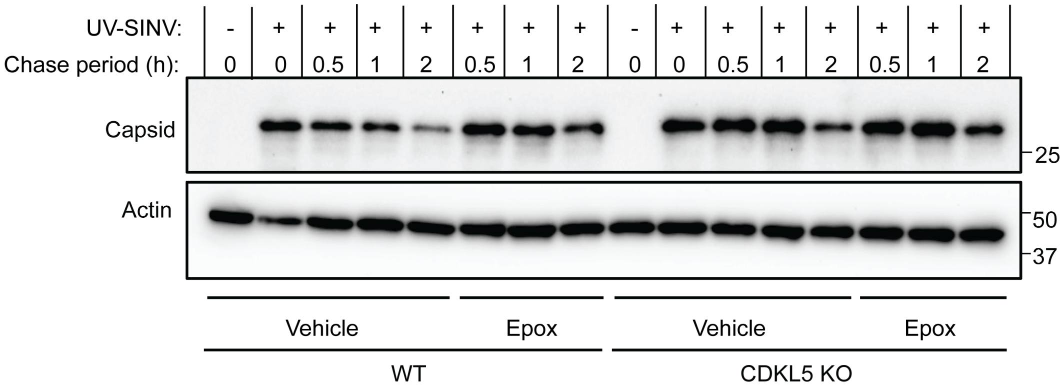

Supplemental Figure 4. Ubiquitin proteasome system partially degrades capsid from UV inactivated SINV

WT and CDKL5 KO HeLa cells exposed to UV-inactivated SINV for 1 h, treated with DMSO (vehicle) or proteasomal inhibitor Epoxomicin (50nM). Capsid degradation detected on immunoblot. Data representative of three independent repeats.

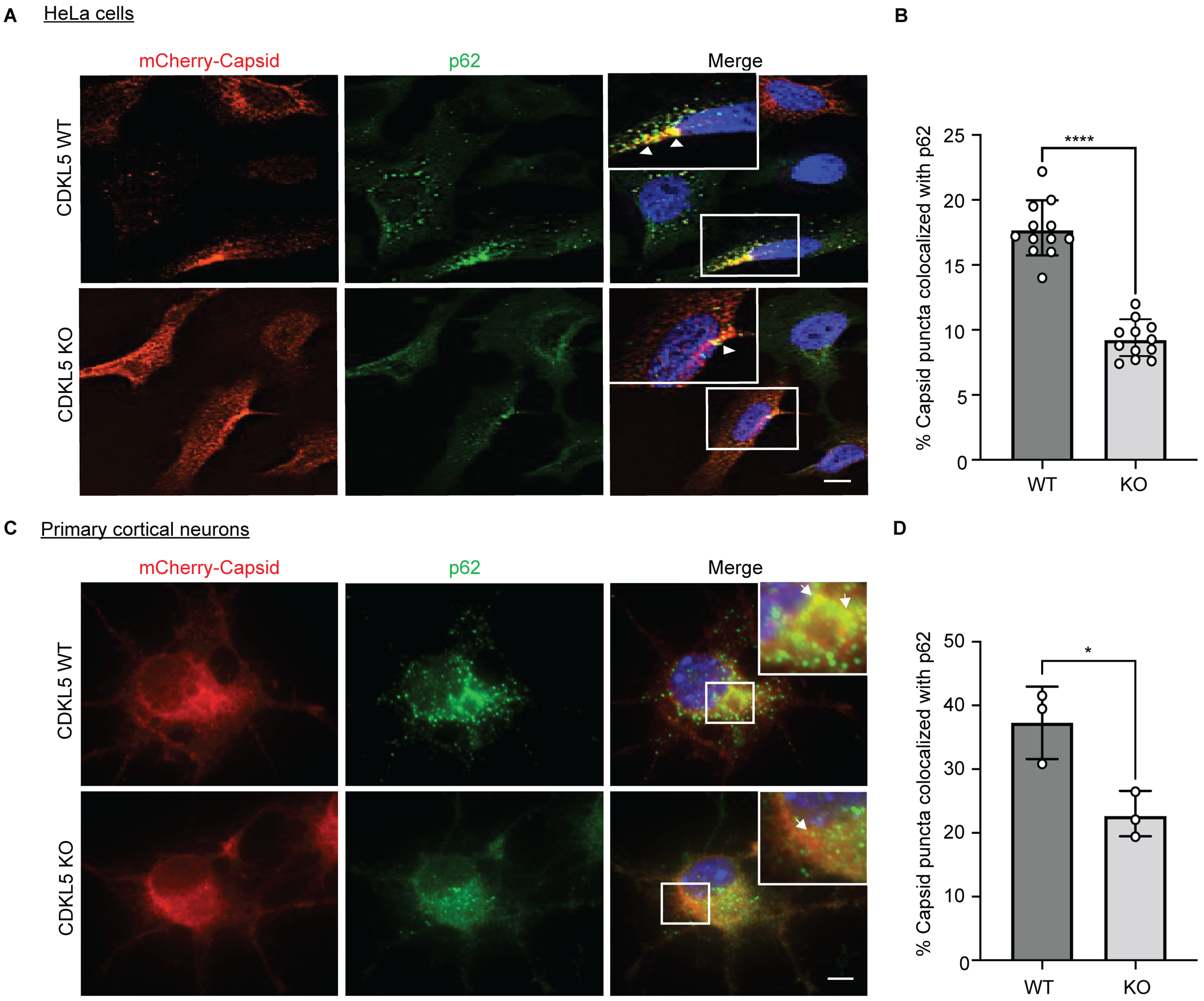

Supplemental Figure 5. CDKL5-deficient cells have defective capsid and p62 interaction
(A,B) WT and CDKL5 KO HeLa cells were infected with SINV/mCherry-capsid (MOI=10, 8h) then stained with antibody to p62 and SINV capsid to boost mCherry capsid visualization. (A) Representative immunofluorescence of p62 and mCherry-capsid with (B) quantification of colocalization. Bars are mean ± SEM of Quantification of percent colocalized mCherry-capsid$^+$ and p62$^+$ puncta with at least 50 cells in triplicate samples from 4 independent experiments analyzed. Arrowheads denote representative colocalized capsid$^+$/p62$^+$ puncta. Scale bar, 10 μm. ****$p < 0.0001$ determined by unpaired two-tailed *t*-test.
(C,D) Primary cortical neurons isolated from littermate CDKL5 WT and CDKL5 KO mice and cultured for 7 days were infected with SINV/mCherry-capsid virus (MOI = 10, 8h) then analyzed through immunofluorescence for mCherry-capsid (red) and p62 (green) colocalization. (A) Representative fluorescent micrographs. Arrows denote mCherry-capsid$^+$/p62$^+$ puncta. Scale bar, 10 μm. (B) Quantification of percent colocalized mCherry-capsid$^+$ and p62$^+$ puncta. Bars are mean ± SEM from neurons isolated from 3 independent embryos per genotype; at least 50 neurons per sample counted. *P* value was determined by unpaired two-tailed *t*-test (*$p < 0.05$).

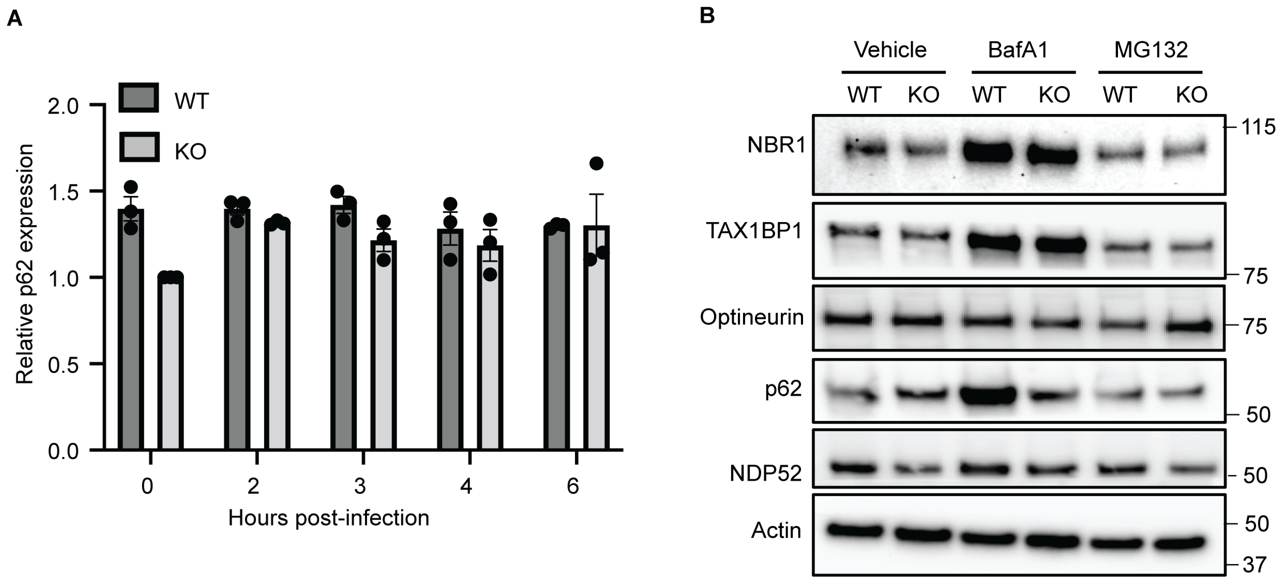

Supplemental Figure 6. p62 expression and selective autophagy protein levels

(A) *p62* expression in WT and CDKL5 KO HeLa cells infected with SINV (MOI = 10) was determined by RT-qPCR normalized to GAPDH. Bars mean ± SEM of triplicate samples and no significance noted on comparisons by unpaired two-tailed t-test; data representative of three independent experiments.

(B) Western blot of autophagy receptors in WT and CDKL5 KO HeLa cells treated with DMSO (vehicle), BafA1 (100 μM, 4h) and the proteasome inhibitor MG132 (10 μM, 8h). Data are representative of two independent experiments.

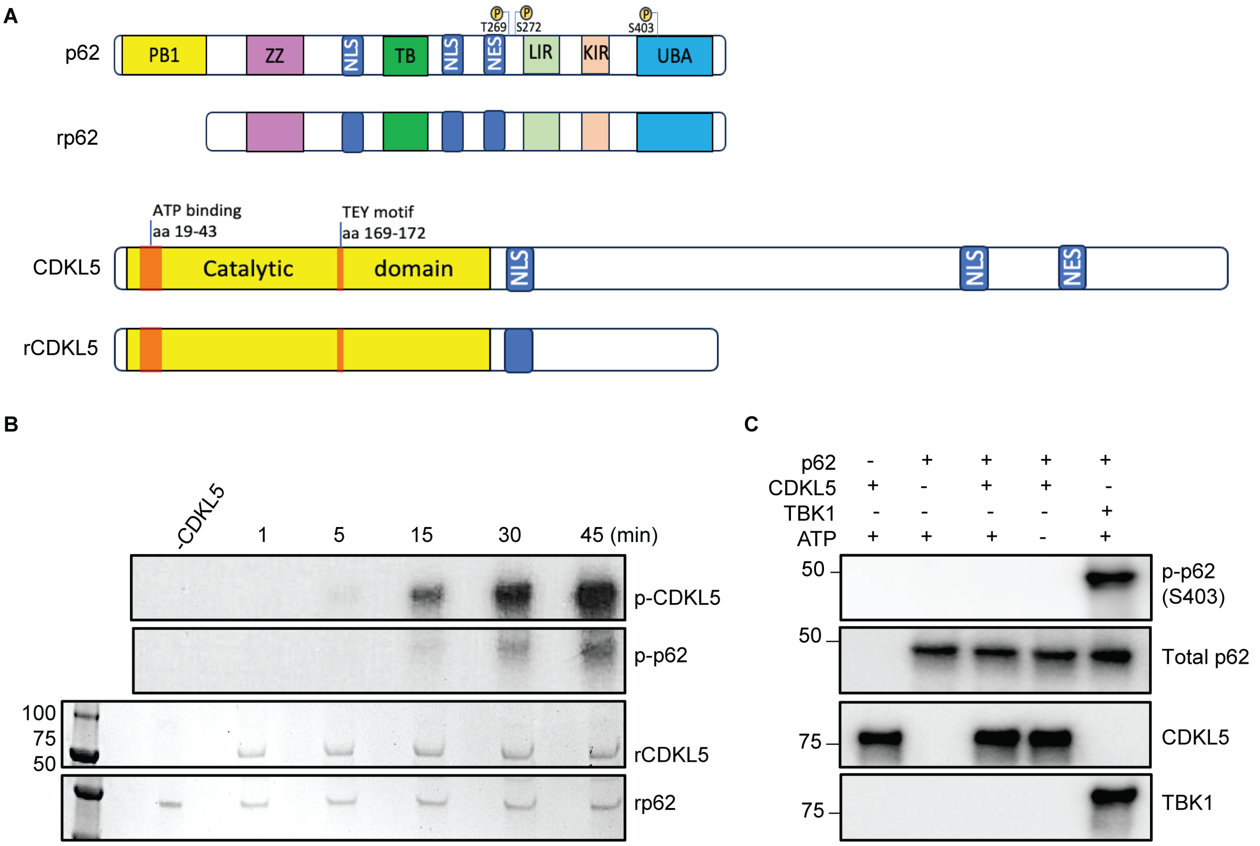

Supplemental Figure 7. Kinetics of CDKL5 phosphorylaton of p62 by in vitro kinase assay

(A) Schematic image of full length p62 and CDKL5 and the recombinant proteins used in the in vitro kinase assay.

(B) In vitro kinase assay with recombinant CDKL5 and p62 was performed using [γ-^{32}P]ATP and the reaction stopped at the indicated time points. The *Top* two panels are autophosphorylation of CDKL5 and phosphorylation of p62 detected by ^{32}P-autoradiography. The *bottom* two panels show Coomassie Brilliant Blue staining of the gel. Blot representative of two independent experiments.

(C) In vitro kinase assay with recombinant CDKL5 (1-498 aa) or TBK-1 and p62 using non-radioactive ATP and detection of p62 phosphorylation by western blot using anti-S403 phosphospecific antibody. Blot representative of two independent experiments

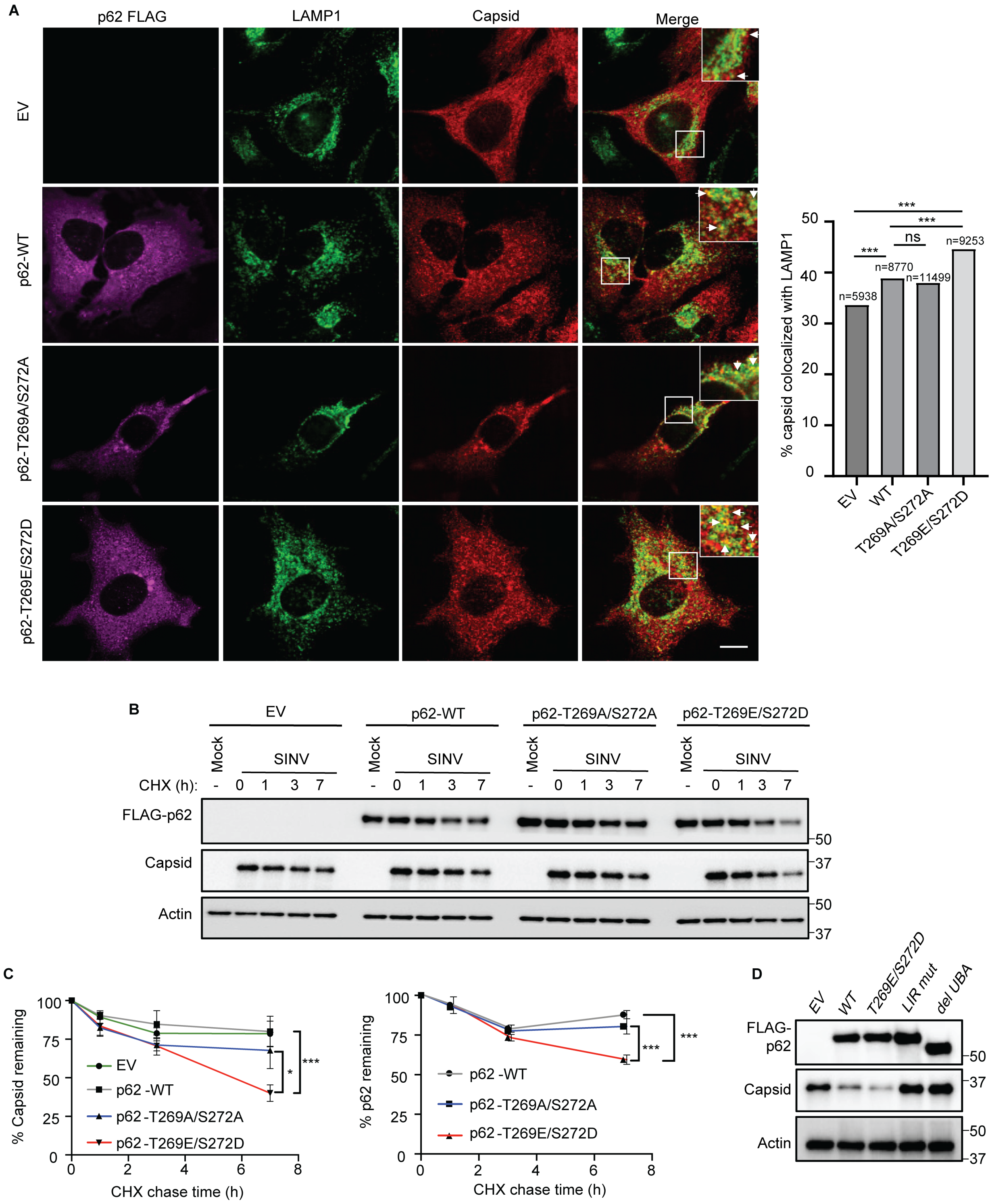

Supplemental Figure 8. CDKL5-mediated p62 phosphorylation facilitates capsid selective autophagy

(A) CDKL5 KO HeLa cells expressing EV, WT p62, p62 with alanine substitution (T269A/S272A) or phosphomimetic p62 (T269E/S272D) tagged with 3X-FLAG were infected

with SINV/HA-capsid virus (MOI 10; 8h) then stained with antibodies to LAMP1, HA for capsid and FLAG for p62 detection. Representative fluorescence micrographs and graph depicting percentage of capsid puncta that colocalize with LAMP1 from the indicated total number of capsid puncta assessed. Over 30 cells per genotype were analyzed through Imaris software. Arrowheads denote representative colocalized puncta. Scale bar, 10 μm. ***$p < 0.001$ determined by chi-square.

(B) The same cell lines from (A) were infected with WT SINV for 6 h then treated with Cycloheximide (CHX) (100ng/ml) for 0, 1, 3, and 7 h. Cell lysates were subjected to immunoblot analysis with the indicated antibodies.

(C) Densitometry quantification of capsid and p62 degradation. Bars are mean ± SEM from three independent experiments. *p* value was determined by two-way ANOVA with Dunnett's multiple comparison test and are significant for the 7 h time point (*$p < 0.05$; ***$p < 0.001$).

(D) Representative immunoblot of capsid accumulation in CDKL5 HeLa cells expressing EV or indicated p62 mutants infected with WT SINV for 8 h.

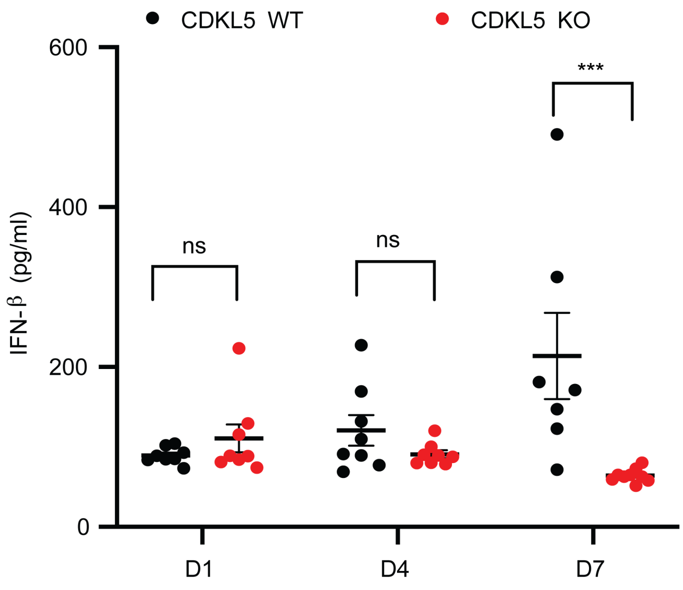

Supplemental Figure 9. CDKL5 modulates in vivo type-1 interferon response during SINV infection

Seven-day-old CDKL5 WT and CDKL5 KO mice were inoculated i.c. with SINV strain dsTE12Q (1 x 10^3pfu). Brains from 7-8 mice per genotype were harvested at the indicated days post infection, homogenized and lysates analyzed for levels of IFN-β through ELISA. Bars represent mean ± SEM of 7-8 mice per group. ***$p < 0.01$ was determined by Mann-Whitney U test.

References:

1. Zufferey R, Nagy D, Mandel RJ, Naldini L, and Trono D. Multiply attenuated lentiviral vector achieves efficient gene delivery in vivo. *Nat Biotechnol.* 1997;15(9):871-5.
2. Naldini L, Blomer U, Gallay P, Ory D, Mulligan R, Gage FH, et al. In vivo gene delivery and stable transduction of nondividing cells by a lentiviral vector. *Science.* 1996;272(5259):263-7.
3. Klionsky DJ, Abdel-Aziz AK, Abdelfatah S, Abdellatif M, Abdoli A, Abel S, et al. Guidelines for the use and interpretation of assays for monitoring autophagy (4th edition). *Autophagy.* 2021;17(1):1-382.
4. Jackson AC, Moench TR, Trapp BD, and Griffin DE. Basis of neurovirulence in Sindbis virus encephalomyelitis of mice. *Lab Invest.* 1988;58(5):503-9.
5. Nabbi A, and Riabowol K. Rapid Isolation of Nuclei from Cells In Vitro. *Cold Spring Harb Protoc.* 2015;2015(8):769-72.
